# Spider mite herbivory induces an ABA-driven stomatal defense

**DOI:** 10.1093/plphys/kiae215

**Published:** 2024-04-26

**Authors:** Irene Rosa-Diaz, James Rowe, Ana Cayuela-Lopez, Vicent Arbona, Isabel Díaz, Alexander M Jones

**Affiliations:** Centro de Biotecnología y Genómica de Plantas (CBGP), Universidad Politécnica de Madrid (UPM)-Instituto Nacional de Investigación y Tecnología Agraria y Alimentaria (INIA/CSIC), Campus de Montegancedo, 20223 Madrid, Spain; Sainsbury Laboratory, Cambridge University, Cambridge CB2 1LR, UK; Confocal Microscopy Unit, Spanish National Cancer Research Center (CNIO), 28029 Madrid, Spain; Departament de Biologia, Bioquímica i Ciències Naturals, Universitat Jaume I, 12071 Castelló de la Plana, Spain; Centro de Biotecnología y Genómica de Plantas (CBGP), Universidad Politécnica de Madrid (UPM)-Instituto Nacional de Investigación y Tecnología Agraria y Alimentaria (INIA/CSIC), Campus de Montegancedo, 20223 Madrid, Spain; Departamento de Biotecnología-Biología Vegetal, Escuela Técnica Superior de Ingeniería Agronómica, Alimentaria y de Biosistemas, UPM, 28040 Madrid, Spain; Sainsbury Laboratory, Cambridge University, Cambridge CB2 1LR, UK

## Abstract

Arthropod herbivory poses a serious threat to crop yield, prompting plants to employ intricate defense mechanisms against pest feeding. The generalist pest 2-spotted spider mite (*Tetranychus urticae*) inflicts rapid damage and remains challenging due to its broad target range. In this study, we explored the Arabidopsis (*Arabidopsis thaliana*) response to *T. urticae* infestation, revealing the induction of abscisic acid (ABA), a hormone typically associated with abiotic stress adaptation, and stomatal closure during water stress. Leveraging a Forster resonance energy transfer (FRET)-based ABA biosensor (nlsABACUS2-400n), we observed elevated ABA levels in various leaf cell types postmite feeding. While ABA's role in pest resistance or susceptibility has been debated, an ABA-deficient mutant exhibited increased mite infestation alongside intact canonical biotic stress signaling, indicating an independent function of ABA in mite defense. We established that ABA-triggered stomatal closure effectively hinders mite feeding and minimizes leaf cell damage through genetic and pharmacological interventions targeting ABA levels, ABA signaling, stomatal aperture, and density. This study underscores the critical interplay between biotic and abiotic stresses in plants, highlighting how the vulnerability to mite infestation arising from open stomata, crucial for transpiration and photosynthesis, reinforces the intricate relationship between these stress types.

## Teaser

*Arabidopsis thaliana* employs abscisic acid–induced stomatal closure as an effective defense mechanism against *Tetranychus urticae* infestation.

## Introduction

Plants and phytophagous arthropods have coevolved in an “arms race” of mutual antagonism between herbivory strategies and plant defenses. Successful feeding by insect and acari pests on compatible crop plants causes substantial yield losses and represents a threat to global food security. Successful plant defense starts in part with the molecular recognition of herbivore-associated molecular patterns (HAMPs) and damage-associated molecular patterns (DAMPs). Through these early warning cues, plants prompt signal transduction pathways and transcriptional reprogramming, resulting in chemical and physical defenses (Stahl et al. 2018; Garcia et al. 2021). This cascade of events requires fine-tuned control mediated by multiple regulatory factors to induce defense regimes appropriate to herbivore species, plant host, and environmental conditions. Common chemical responses to herbivore damage include the accumulation of reactive oxygen species (ROS), calcium, and several hormones considered to be core modulators of immune signaling, i.e. jasmonic acid (JA), jasmonoyl-isoleucine (JA-Ile), and salicylic acid (SA; Erb and Reymond 2019). These responses are also shared with many plant–pathogen interactions (Bari and Jones 2009). Physical defenses against pests vary considerably; for example, lignified and suberized root and stem barriers, aerial trichomes, and epidermal cuticles can all restrict access to feeding (War et al. 2012; Holbein et al. 2019). Induced closure of stomata, as natural entry sites to the leaf interior, has received considerable attention for foliar pathogen responses (Melotto et al. 2008, 2017). On the other hand, pathogen-induced closure of stomata can also contribute to water soaking and microbial pathogenesis later in infection (Hu et al. 2022; Lajeunesse et al. 2023). Stomatal closure has also been observed in response to phytophagous arthropods (Sances et al. 1979; Pincebourde and Casas 2006; Schmidt et al. 2009), though whether closure constituted a defense mechanism or contributed to the infestation remained unclear.

Abscisic acid (ABA) is the key hormone regulating stomatal aperture, which gates gas exchange for transpiration, photosynthesis, and responses to abiotic stimuli (Kuromori et al. 2018) and is accumulated in response to a number of environmental stimuli, including drought, salt, and wounding (Christmann et al. 2006). Both chewing and piercing–sucking arthropod pests provoke loss of water, an increase in transpiration, and a reduction in stomata conductance. All these phenomena are associated with water stress–induced accumulation of ABA and subsequent stomatal closure (Lim et al. 2015). ABA-deficient mutants are more susceptible to chewing herbivory by beet armyworm (*Spodoptera exigua*; Thaler and Bostock 2004) and African cotton leafworm (*Spodoptera littoralis*) caterpillars (Bodenhausen and Reymond 2007), suggesting ABA signaling and potentially stomatal closure could contribute to plant defense. Open stomata are natural apertures where piercing–sucking herbivores like aphids and mites insert their stylets, specialized feeding structures, to access nutrient-rich subepidermal cells. Although stylets can also be inserted between epidermal cells, feeding via opening stomata is potentially faster and avoids the damage of cell walls, which can trigger rapid plant defenses (Mathen et al. 1988; Bensoussan et al. 2016). In some cases, stomata are even preferred places to lay herbivore eggs (DeClerck and Steeves 1988). On the other hand, the caterpillar *Helicoverpa zea* was found to actively induce stomatal closure to reduce the release of herbivore-induced plant volatiles that can recruit *H. vea* predators (Lin et al. 2021). More direct regulation of ABA by piercing–sucking herbivores is also possible as the saliva of the aphid *Myzus persicae* upregulated genes that also responded to ABA treatment (Hillwig et al. 2016). In this interaction, ABA contributes to susceptibility to aphid infestation, indicating that the role of ABA signaling in herbivory defense could also be negative.

ABA is perceived by PYRABACTIN RESISTANCE (PYR)/PYR-like (PYL) or REGULATORY COMPONENT OF ABA RECEPTOR (RCAR) family receptor proteins (Gonzalez-Guzman et al. 2012). The accumulation of ABA in stomatal guard cells, for example, during water stress, is transduced via PYR/PYL/RCAR receptors to a complex signaling network (Ma et al. 2009). Core components include inhibitory PROTEIN PHOSPHATASE 2C (PP2C)-type proteins as well as positive regulators of ABA signaling including SUCROSE NON-FERMENTING 1-RELATED PROTEIN KINASE 2 (SnRK2)-type protein kinases as well as ABRE-BINDING FACTOR (ABF) and ABA Insensitive (ABI)-type transcription factors (Hsu et al. 2021). ABA effects comprise changes in redox homeostasis, guard cell–specific kinase regulation, and cytosolic Ca^2+^ levels. These events lead to stomatal closure while regulating numerous nonstomatal functions (Neill et al. 2008; Dinh et al. 2013; Ng et al. 2014). ABA regulation of these components also occurs in many plant–pathogen interactions, and as for plant–herbivore interactions, ABA has been found to both promote and antagonize microbial defenses (Bari and Jones 2009; Lim et al. 2015).

One explanation for contrasting roles for ABA in diverse plant defense responses is ABA crosstalk with ROS, calcium, and defense hormone signaling, for example, through inhibition of SA signaling (De Torres-Zabala et al. 2007; Ton et al. 2009). ABA activation of primed JA-regulated defense responses in Arabidopsis (*Arabidopsis thaliana*) contributes to induced resistance against small white (*Pieris rapae*) caterpillar herbivory (Vos et al. 2013). ABA can also regulate JA responses via direct interaction of PYL5/6 ABA receptors with MYC transcription factors (Aleman et al. 2016), and ABA amplifies JA-dependent defense responses as part of the signal transduction pathway in plants elicited by oral secretions of insects (Dinh et al. 2013). Thus, ABA-JA and ABA-SA hormonal crosstalk could also be important for ABA responses in the plant–herbivore interplay. With respect to stomatal aperture, SA and methyl-JA can promote stomatal closure while other jasmonates, particularly the bioactive JA-Ile, can induce stomatal opening (Munemasa et al. 2011), though the contribution of SA and JA signaling might be secondary to ABA signaling (Zamora et al. 2021).

Most reports on the interaction between plants and arthropod herbivores have focused on insects, but mites are also highly economically relevant agricultural pests (Vacante 2016). Among them, the 2-spotted spider mite (*Tetranychus urticae*), an acari of the Tetranychidae family, is one of the most polyphagous pests found worldwide, feeding on more than 1,100 documented host plants, of which about 150 are crops (Migeon and Dorkeld 2023). *T. urticae* pierces individual parenchymatic cells with stylets specialized for sucking their content. Infestation with *T. urticae* causes leaf chlorosis, and severe infestation causes substantial crop losses (Bensoussan et al. 2016). In addition to its economic importance, *T. urticae* has a sequenced genome and a number of available tools and protocols (Grbić et al. 2011; Cazaux et al. 2014; Suzuki et al. 2017; Ojeda-Martinez et al. 2020). These characteristics and its ability to feed on the *A. thaliana* model plant led to *T. urticae* becoming a model herbivore for plant–pest interactions. However, potential positive or negative roles for ABA accumulation or signaling, including stomatal closure, in the context of plant defense against mite herbivores remain largely unexamined. How this key plant hormone that integrates a myriad of environmental cues impacts a generalist plant herbivore is relevant for a wider understanding of the plant–pest interplay.

Here, we have analyzed the capacity of *T. urticae* to trigger stomata closure at the feeding site and on nearby undamaged tissues using *A. thaliana* as a host plant. We also studied stomatal behavior and hormone crosstalk during mite infestation. We have demonstrated the accumulation of ABA and characterized this accumulation at cellular resolution using the nuclear-localized Forster resonance energy transfer (FRET) biosensor for ABA nlsABACUS2-400n (Rowe et al. 2023). Mite feeding bioassays in mutants for ABA production and perception, as well as in lines with altered stomata density, have established the biological relevance of stomatal control, which are entry gates for mite stylets, for plant defenses in the plant–mite interaction.

## Results

### *T. urticae* infestation induces stomata closure and modifies hormone content

**Figure 1.**
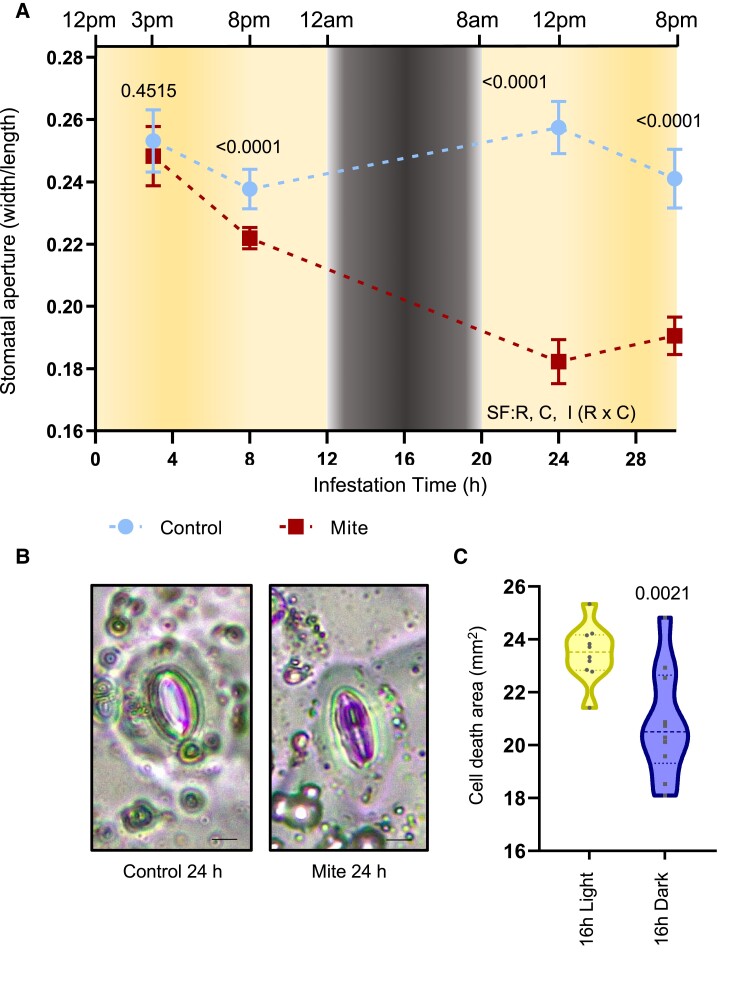
Effects of mite infestation on stomata aperture and on the leaf cell death when plants are incubated under light or dark conditions. **A)** Stomatal aperture measured in Arabidopsis Col-0 detached leaves after 3, 8, 24, and 30 h of mite infestation. Results referred as width/length ratio. Significant factors (SF) indicate whether the 2 independent factors, R (infestation time) and C (mite treatment), and/or their interaction I (R × C) were statistically significant (two-way ANOVA followed by Sidak's multiple comparison test, *P* < 0.05). Detailed ANOVA results are available in Supplementary Table S5. Numbers indicate significant differences between control and mite treatment at each point of infestation. Data are means ± Se of 28 biological replicates. **B)** Image of stomata closure 24 h after mite infestation. Bars = 8 *µ*m. **C)** Cell death, measured in leaf disks by trypan blue staining after 16 h of mite infestation under dark and light conditions, is expressed in square millimeters. A two-tailed *t* test was used to test differences due to light treatments (*P* < 0.05). Data are means ± Se of 10 biological replicates.

To study time-resolved dynamics of defense and stomatal regulation upon mite infestation, the stomatal aperture on the abaxial leaf surface was analyzed in epidermal samples at four infestation time points (Fig. 1A). Leaf epidermal impressions showed open stomata during the day with subtle reductions in aperture in wild-type (WT) Col-0 noninfested plants toward the end of the light period (Fig. 1A, Supplementary Table S1). Mite infestation induced a striking stomatal closure, reaching the maximum closure at 24 to 30 h postinfestation (Fig. 1, A and B). To explore whether natural stomatal closure in darkness could alter mite feeding habits and resultant leaf cell death, plants were grown in regular conditions, infested, and incubated under a constant dark or light environment for 16 h, and then cell damage was assayed. Results demonstrated that mites fed better under light conditions, when stomata are open, as plants suffered increased cell death (Fig. 1C). Nonetheless, this could also be due to other light-driven changes, such as plant hormone, carbohydrate, ROS and calcium levels, or changes in mite behavior.

**Figure 2.**
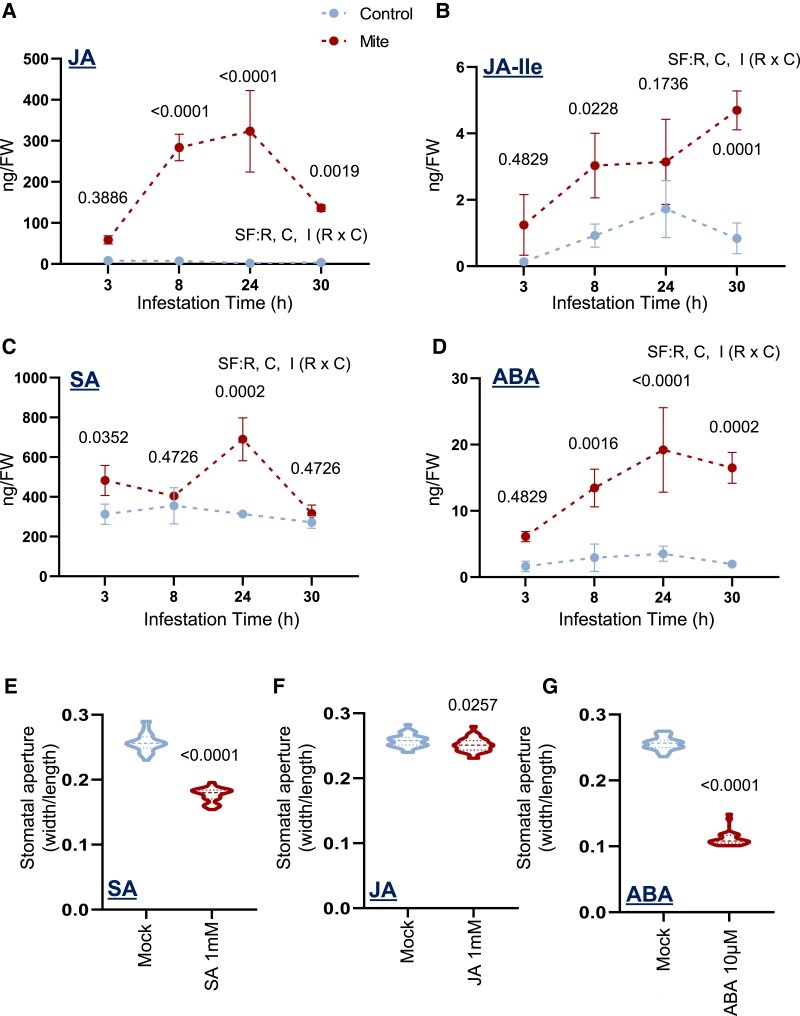
Quantification of hormone content in Arabidopsis Col-0 upon mite infestation and stomata aperture after exogenous hormonal treatments. **A)** JA, **B)** JA-Ile, **C)** SA, and **D)** ABA accumulation in whole plants was quantified at 3, 8, 24, and 30 h after mite infestation. Values are expressed as nanograms of hormone per gram of fresh weight (FW). Significant factors (SF) indicate whether the 2 independent factors, R (infestation time) and C (mite treatment), and/or their interaction I (R × C) were statistically significant (two-way ANOVA followed by Tukey's test, *P* < 0.05). Detailed ANOVA results are available in Supplementary Table S5. Numbers indicate significant differences compared to control conditions. Data are means ± Se of 3 pools of 6 biological replicates. Stomata aperture measured in detached leaves at 3 h after spraying on the aerial part of the plant with **E)** 1 mM SA, **F)** 1 mM JA, and **G)** 10 *µ*M ABA. Results represented as width/length ratio. Student’s *t* test was used to assess differences due to hormone treatments (*P* < 0.05). Data are means ± Se of 10 biological replicates.

Since hormone accumulation in response to mites might be responsible for phenotypic changes in stomatal behavior, SA, JA, JA-Ile, and ABA were quantified in mite-infested Arabidopsis Col-0 leaves at 4 postinfestation time points. The accumulation of all 4 compounds was induced by mites but with distinct temporal profiles. SA content significantly increased from the earliest infestation time, while JA, JA-Ile, and ABA required longer times to be differentially accumulated in comparison to noninfested plants (Fig. 2, A to D). The highest hormone levels were detected at 24 h of infestation, except for the JA-Ile, which reached the highest content at 30 h of infestation, in accordance with a reduction in its JA precursor at this time point. Interestingly, ABA levels were maintained at 30 h, while SA was reduced. To determine the impact of SA, JA, and ABA on leaf stomatal aperture, these hormones were exogenously applied to Arabidopsis Col-0 plants. SA and ABA treatment triggered stomatal closure in treated leaves, as expected (effect size in Supplementary Table S2), though the effect of JA was negligible (Fig. 2, E to G).

### ABA accumulation was detected upon mite infestation

**Figure 3.**
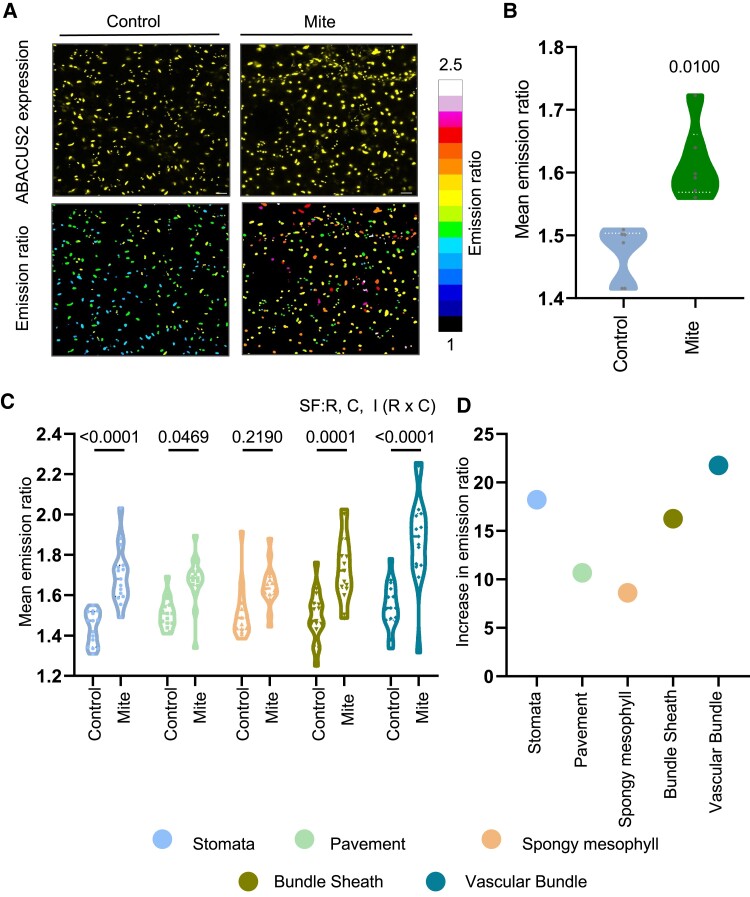
ABA accumulation at the cellular resolution in leaf tissues using the nuclear ABA biosensor plants nlsABACUS2-400n, after mite infestation. **A)** Z-Projections of analyzed images. Upper images correspond to nuclei expressing the biosensor and bottom images to FRET emission ratios in detached leaves. Black areas represent areas excluded from analysis during segmentation. **B)** Quantification of nlsABACUS2-400n emission ratios in multiple leaves. Student’s *t* test was used to assess differences due to control and mite conditions (*P* < 0.05). Data are means of 8 biological replicates. **C)** Emission ratios of nlsABACUS2-400n biosensor in cells of stomatal, pavement, spongy mesophyll, bundle sheath, and vascular bundle cells, after 24 h of mite infestation in detached leaves. Significant factors (SF) indicate whether the 2 independent factors, R (mite treatment) and C (nucleus type), and/or their interaction I (R × C) were statistically significant (two-way ANOVA followed by Tukey's test, *P* < 0.05). Detailed ANOVA results are available in Supplementary Table S5. Data are means of 15 biological replicates. **D)** Increased levels of emission ratio in the different 5 cell types after infestation when compared to noninfested plants.

Since ABA accumulates in leaves during mite infestation concomitantly with stomatal closure, we sought to identify in which cells ABA could be functionally relevant for Arabidopsis defense using the high-resolution nlsABACUS2-400n FRET-based biosensor for ABA (Rowe et al. 2023). In leaves of Arabidopsis plants expressing the biosensor, nuclei of epidermal and internal cell types showed sufficient biosensor expression for segmentation (Fig. 3A, upper images). In all detected cell types, nuclear ABACUS2-400n fluorescence emission ratios were higher in infested plants than in controls (Fig. 3, A, bottom images, and B), indicating higher ABA concentrations. Mean emission ratio for all cells was significantly higher for mite-infested leaf images (Fig. 3B). To better understand the cellular distribution of ABA accumulation, we used a nuclear morphology classifier based on the “FRETENATOR” tool to analyze cell type emission ratios. Nuclei were classified into 5 cell types: stomata, pavement, spongy mesophyll, bundle sheath, and vascular bundle cells. With infestation, the signal increased in all cell type groups, but stomatal and vascular bundle cell groups showed the highest emission ratios (Fig. 3, C and D).

Because *T. urticae* cause cell death by inserting their stylets either in between epidermal pavement cells or through stomatal pores to suck mesophyll cell contents (Supplementary Fig. S1A; Bensoussan et al. 2016), it was important to get information about the viability status of leaf cells after the infestation. The ABA biosensor allowed us to detect cell viability since nuclei detected by biosensor fluorescence reflect viable cells. The comparison between infested and noninfested plants showed significant changes in the total number of quantified nuclei (Supplementary Figs. S1B and S2A). However, when this comparison was independently done in each of the 5 cell type groups, the mesophyll cell type presented a lower number of nuclei in the infested plants than in noninfested (Supplementary Fig. S1, C to G). Taken together, these results corroborated that spongy mesophyll cells were the target tissue where mites suck nutrients upon their feeding and indicated that nlsABACUS2-400n biosensor was able to detect local ABA increases in response to mites in leaf cell types directly involved in feeding as well as in distal cells and cell types not directly involved in feeding, i.e. bundle sheath and vascular cells. Interestingly, vascular cells have been proposed to be the major sites of foliar ABA biosynthesis (Endo et al. 2008), and thus, our data provide support for a model in which mite feeding triggers ABA synthesis in the vasculature and this results in the translocation of ABA across the leaf cell types.

### ABA is involved in plant defense against mites

**Figure 4.**
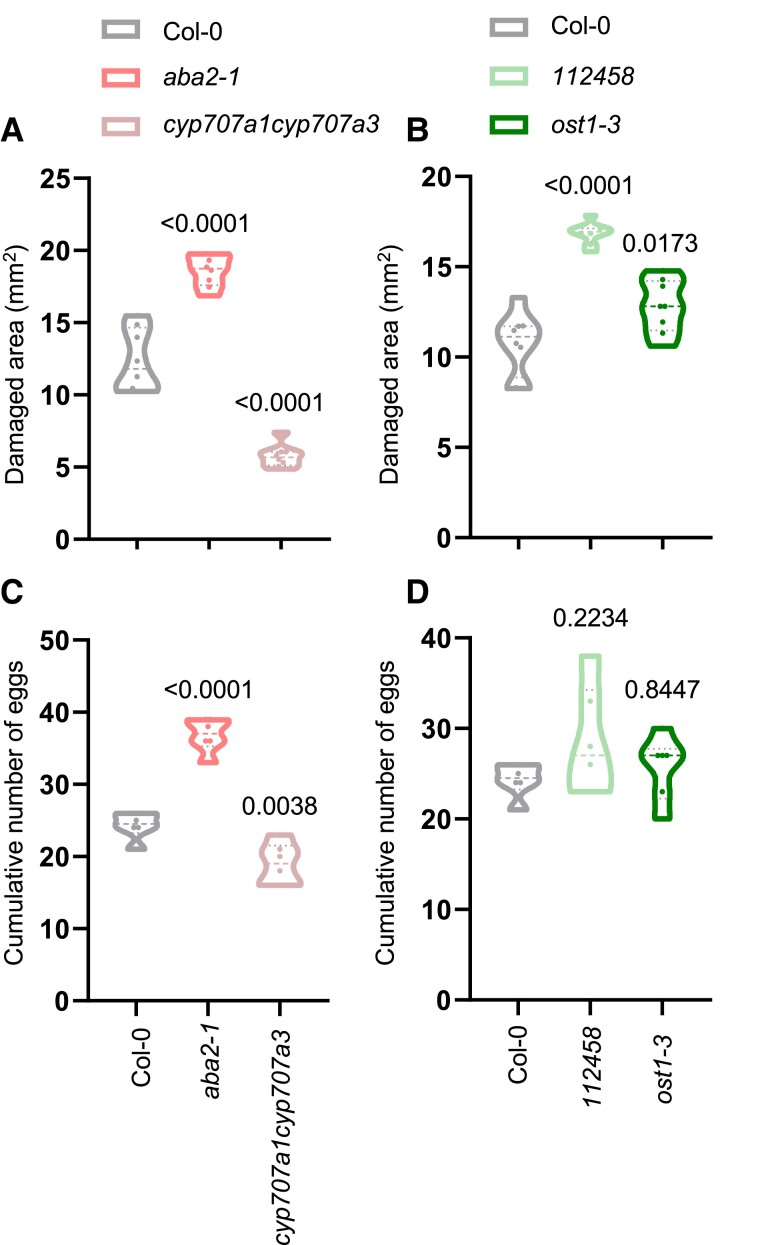
Plant damage and mite fecundity after mite infestation of *aba2-1* and *cyp707a1cyp701a3* and *pyr1pyl-112458*, *ost1-3* Arabidopsis mutants, and Col-0 plants. **A)** Foliar damage quantified 4 d after mite infestation in whole plants in *aba2-1*, *cyp707a1cyp707a3*, and Col-0 plants and in **B)***112458*, *ost1-3*, and Col-0 plants. Data are expressed in square millimeters. Effects on *T. urticae* fecundity measured 36 h after the infestation with synchronized mite females in detached leaves on **C)***aba2-1 cyp707a1cyp707a3* and Col-0 plants and in **D)***112458*, *ost1-3*, and Col-0 plants. Numbers indicate significant differences compare to Col-0 genotype. Data are means for 10 biological replicates for damage area and 6 biological replicates for eggs. **A to D)** One-way ANOVA followed by Tukey's multiple comparisons test, *P* < 0.05. Detailed ANOVA results are available in Supplementary Table S5.

To study how changes in endogenous ABA levels affect mite infestation, Arabidopsis mutants in ABA biosynthesis and catabolism, *aba2-1* and *cyp707a1cyp707a3*, respectively, which result in lower and higher ABA concentrations (González-Guzmán et al. 2002; Okamoto et al. 2009), were selected to perform mite infestation. *aba2-1* lines showed significantly greater damage than control plants upon mite feeding for 4 d while the damaged area in *cyp707a1cyp707a3* line was lower than in Col-0 plants (Fig. 4A). Likewise, fecundity rates, measured as cumulative number of eggs after 36 h, were higher when mites fed on the mutant *aba2-1* mutant line than when they fed on Col-0 plants and were significantly reduced in the *cyp707a1cyp707a3* line (Fig. 4C). These data indicated that ABA promotes plant defenses against mite damage and antagonizes mite oviposition. Additionally, 2 Arabidopsis ABA insensitive mutants, the *112458* ABA receptor sextuple mutant and *ost1-3* SnRK2 kinase mutant, were also examined to determine if the canonical ABA signaling pathway participated in ABA-dependent mite responses. The OST1 gene is preferentially expressed in guard cells and the vasculature (Mustilli et al. 2002), cell types that exhibited the most pronounced increase in ABA levels (Fig. 3). While mite females laid a similar number of eggs in mutants as in control plants, both mutant lines showed more damage than Col-0 plants after infestation (Fig. 4, B and D). Overall, these results revealed that ABA signaling, including in the vasculature or stomata, is important for mite defense.

Because all cells respond with increased ABA, we sought to determine whether ABA accumulation further impacted SA and JA, the core hormones involved in plant defenses. We examined the expression of *PR1* and *MYC2*, marker genes of SA and JA signaling, respectively, in the 5 Arabidopsis genotypes at 2 infestation times. The expression of the *PR1* gene was induced at 8 h postinfestation and decreased 24 h after mite feeding in all studied genotypes. However, *PR1* levels were lower in the *aba2-1* line and higher in the *cyp707a1cyp707a3* line than in Col-0 plants at the 8-h time point and displayed the opposite expression pattern at 24 h (Supplementary Fig. S3A). No differences in *PR1* behavior were found between *112458*, *ost1-3* mutants, and WT plants (Supplementary Fig. S3B). Together, these results indicate that ABA could crosstalk with SA signaling positively early in mite infection and negatively later in infection. In contrast to *PR1*, which peaked at 8 h, *MYC2* presented the maximum induction at 24 h after infestation (Supplementary Fig. S3, C and D). In the 5 tested genotypes, only the *aba2-1* mutant line showed altered MYC2 expression, i.e. elevated at 24 h (Supplementary Fig. S3, C and D). The observation that the reduced ABA levels in the *aba2-1* mutant increased expression of both *PR1* and *MYC* genes at 24 h could indicate hormone crosstalk but could also be an indirect result of increased mite infestation in *aba2-1*. Certainly, the overaccumulation of *MYC2* transcript suggests that positive crosstalk of ABA with JA signaling is unlikely to explain the role of ABA in mite resistance.

One physiological response associated with ABA is the production of ROS, particularly H_2_O_2_, a signaling molecule that regulates plant stress response genes (Li et al. 2022). To further confirm such an association, the H_2_O_2_ content was determined in detached leaves of the 4 Arabidopsis mutants and Col-0 plants after 24 h of mite infestation (Supplementary Fig. S4). The measurement of H_2_O_2_ concentration is represented as relative DAB staining units (Supplementary Fig. S4A) and demonstrated that *aba2-1* and *cyp707a1cyp707a3* infested lines accumulated higher and lower levels of H_2_O_2_, respectively, than Col-0 plants. In contrast, the number of DAB deposits in the *ost1-3* mutant was only slightly reduced and was not altered in *112458* after mite feeding (Supplementary Fig. S4). As with JA signaling, a negative association between ABA levels and ROS production suggests ROS accumulation is unlikely to explain the role of ABA in resistance to mite infestation.

### The effects of exogenous ABA application on mite infestation

**Figure 5.**
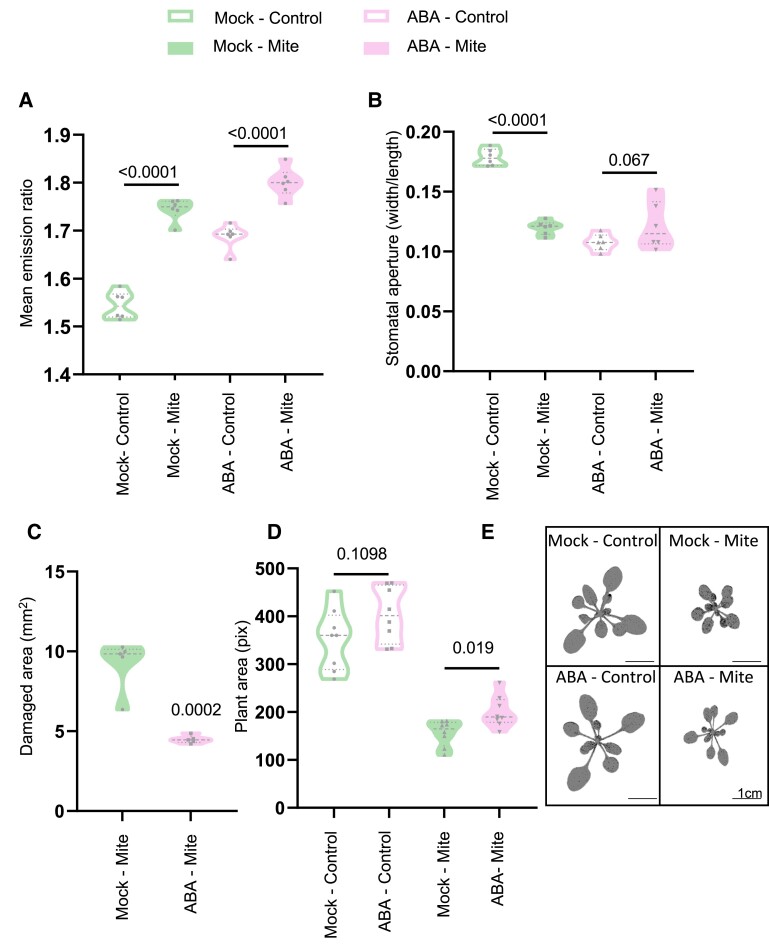
Effect of ABA pretreatment of nlsABACUS2-400n plants before mite infestation. **A)** Emission ratio of biosensor signal for detached leaves under combined 3 h ABA pretreatment and 24 h infestation or controls; *t* tests with a false discovery rate multiple testing correction were used. Numeric annotations represent *q*-values, *n* = 6. **B)** Stomatal aperture presented as the width/length ratio in detached leaves under 3 h ABA pretreatment and 24 h infestation or controls; *t* tests with a false discovery rate multiple testing correction were used. Numeric annotations represent *q*-values, *n* = 6. **C)** Damaged area in whole plants expressed in square millimeters with and without 3 h ABA pretreatment and 24 h infestation or controls. Student’s *t* test was used. Number annotation represents *P*-values, *n* = 5. **D)** Whole plant growth quantified in pixels; *t* tests with a false discovery rate multiple testing correction were used. Number annotations represent *q*-values, *n* = 10. **E)** Infested Arabidopsis plant images of either 3 h ABA pretreatment and 24 h infestation or controls. Images show the digitally extracted plant rosettes used for quantification in **D)**.

As demonstrated above, exogenous ABA application induces stomata closure (Fig. 2G). To elucidate if ABA treatment affected plant defenses, we pretreated nlsABACUS2-400n plants by spraying ABA on the leaves 3 h before mite infestation and analyzed cellular ABA levels and leaf responses to mites. As expected, ABA pretreated and mite-infested plants displayed higher nlsABACUS2-400n biosensor emission ratios, indicating higher ABA levels, and the highest emission ratios were observed in those plants that were first pretreated and then infested (Fig. 5A). Both ABA pretreatment and mite infestation triggered stomata closure, with no synergistic or additive effects observed when the treatments were combined (Fig. 5B). The exogenous application of ABA before mite infestation helped plant defenses since they showed less damage than nonpretreated plants and provided positive effects on plant growth, as shown in measurements of the rosette area (Fig. 5, C to E). Thus, ABA pretreatment enhanced mite defenses and favored plant growth during infestation.

### Stomatal aperture determines the mite infestation outcome

**Figure 6.**
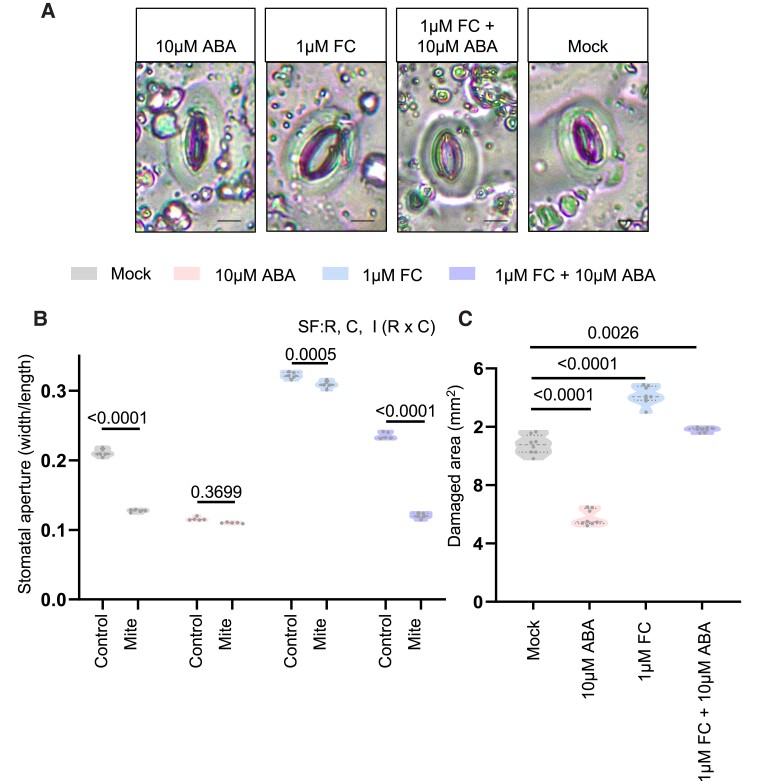
Effects of 10 *µ*M ABA and/or 1 *µ*M FC pretreatments on stomatal aperture and on Arabidopsis plant responses to mite infestation. **A)** Images of stomata behavior in ABA and/or FC pretreated plants. Bars = 8 *µ*m. **B)** Stomatal aperture in ABA and/or FC pretreated plants after 24 h of mite infestation in detached leaves. Significant factors (SF) indicate whether the 2 independent factors, R (mite treatment) and C (pretreatment), and/or their interaction I (R × C) were statistically significant (two-way ANOVA followed by Tukey's multiple comparison test, *P* < 0.05). Detailed ANOVA results are available in Supplementary Table S5. Data are means of 5 biological replicates. **C)** Plant damage in whole plant of ABA and/or FC pretreated plants after 4 d of mite infestation. Numbers indicate significant differences compared to mock treatment. One-way ANOVA followed by Tukey's multiple comparison test, *P* < 0.05. Detailed ANOVA results are available in Supplementary Table S5. Data are means of 8 biological replicates.

As ABA accumulation and stomata closure are induced in response to mites, we questioned whether increased stomata aperture could provide any advantage for mite infestation. To clarify this point, Col-0 plants were pretreated with fusicoccin (FC), a compound that promotes stomata opening (Hunt et al. 2010), with ABA to close stomata, or both. After treatments, stomatal aperture and plant damage were measured, and results were compared with mock-treated and noninfested plants. Plants pretreated either with ABA or FC presented closed and opened stomata, respectively, and the combination of both produced a stomatal aperture comparable to mock plants (Fig. 6, A and B). An experiment using different FC + ABA ratios demonstrated that very low concentrations of FC (0.5 *µ*M) were sufficient to keep stomata open (Supplementary Fig. S5). Upon mite infestation, stomata remained closed in ABA-treated plants, remained open in FC-treated plants, and were actively closed similarly to mock when ABA and FC were previously combined (Fig. 6B). Plant damage results showed that more open stomata in the FC treatment correlated with higher leaf damaged area, indicating that open stomata facilitated mite feeding (Fig. 6C). As leaf damage increased in plants subjected to combined ABA and FC treatments compared with the ABA treatment alone, the control over stomatal aperture function of ABA is likely key for resistance to mite infestation.

### Stomatal density affects mite infestation

**Figure 7.**
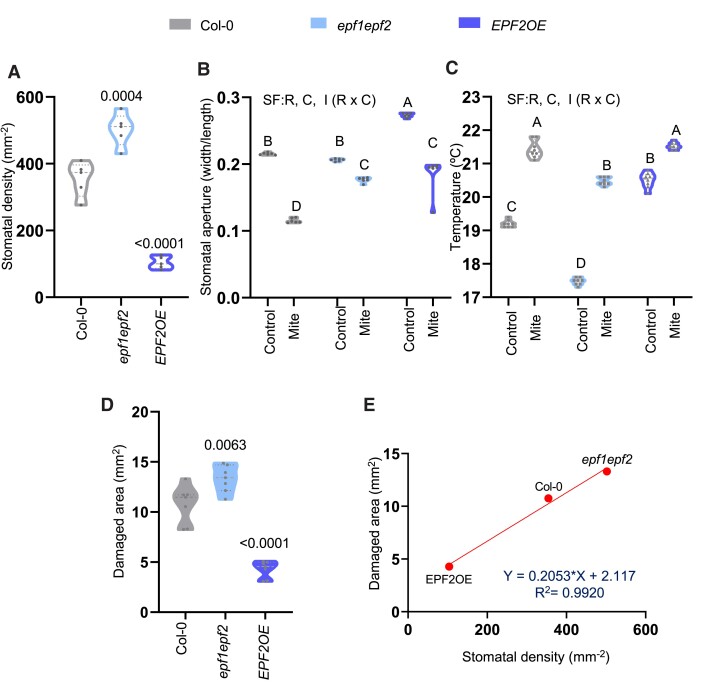
Stomatal density in epf1epf2, an EPF2 overexpressing line (EPF2OE), and Col-0 plants and determination of damage, stomata aperture, and temperature in the 3 genotypes after mite infestation. **A)** Stomatal density of Col-0, *epf1epf2*, and *EPF2OE* expressed per square millimeter from detached leaves. Numbers indicate significant differences compared to Col-0 genotype. Data are means of 5 biological replicates. One-way ANOVA followed by Tukey's multiple comparisons test, *P* < 0.05. Detailed ANOVA results are available in Supplementary Table S5. **B)** Stomatal aperture represented as width/length ratio in detached leaves of Col-0, *epf1epf2*, and *EPF2OE* under mite infestation or control. Data are means of 6 biological replicates. One-way ANOVA followed by Tukey's multiple comparisons test, *P* < 0.05. Detailed ANOVA results are available in Supplementary Table S5. **C)** Temperature in degree Celsius measured in whole plants of Col-0, *epf1epf2*, and *EPF2OE* under mite infestation or control. Significant factors (SF) indicate whether the 2 independent factors, R (mite treatment) and C (genotype), and/or their interaction I (R × C) were statistically significant (two-way ANOVA followed by Tukey's multiple comparison test, *P* < 0.05). Detailed ANOVA results are available in Supplementary Table S5. Data are means of 6 biological replicates. **D)** Foliar damage in whole plant, expressed in square millimeters, was quantified 4 d after mite infestation of Col-0, *epf1epf2*, and *EPF2OE*. Data are means of 7 biological replicates. One-way ANOVA followed by Tukey's multiple comparisons test, *P* < 0.05. Detailed ANOVA results are available in Supplementary Table S5. **E)** Correlation between stomatal density and damaged area for the 3 Arabidopsis genotypes (Pearson’s product moment *R*^2^ = 0.992).

Given the importance of stomatal closure as a defense mechanism against mite infestation, we investigated the potential involvement of stomatal density in the leaf as a contributing factor using Arabidopsis lines with higher and lower numbers of stomata than Col-0 plants. The selected plants were mutants in EPIDERMAL PATTERNING FACTOR (EPF), a family of secreted peptides that inhibit stomatal development (Hepworth et al. 2015). In particular, we selected the *epf1epf2* mutant line and EPF2OE, an EPF2 overexpressing line. First, stomatal density was measured prior to mite infestation (Fig. 7A), and as expected, the *epf1epf2* mutant exhibited an elevated stomatal count, while the EFF2OE line displayed a reduced number of stomata. Then, we evaluated stomatal aperture in the 3 Arabidopsis genotypes with and without mite infestation and found that stomatal aperture in EPF2OE plants was higher than in *epf1epf2* or Col-0 plants (Fig. 7B). Interestingly, infested *epf1epf2* mutant exhibited somewhat lessened stomatal closure than Col-0, though infestation closed stomata in all genotypes (Fig. 7B). As stomata allow gas exchange between leaf mesophyll cells and atmosphere, contributing to leaf cooling (Chowdhury et al. 2021), we also analyzed whether the leaf temperature was dependent on the stomata number and if temperature variations were produced during mite infestation. Uninfested temperature of *epf1epf2* leaves was 2.2°C less than Col-0 and 3.1°C less than EPF2OE leaves (Supplementary Table S3, Fig. 7C), consistent with stomatal density measurements. After mite infestation, an expected increase in leaf temperature was detected in Col-0 (+2.2°C) and, to a lesser extent, in EPF2OE (+1°C) plants that have fewer stomata. Mite infestation augmented leaf temperature in *epf1epf2* (+3.0°C), the mutant line with more stomata, although infested leaves remained cooler than in infested Col-0 and EPF2OE (Supplementary Table S3, Fig. 7C). These temperature data corroborated the interrelationship between stomatal aperture and density in the plant response to mite infestation.

Finally, the damage produced by mites (Fig. 7D) was quantified in these Arabidopsis plants. The *epf1epf2* line displayed more damage than Col-0 plants, while mite damage was significantly reduced in the EPF2OE line. The close correlation between stomatal density and damage (*R*^2^ = 0.9920; Fig. 7E) indicates that the density of stomata in leaves is an essential feature that determines the success of mite infestation.

## Discussion

Stomata are specialized microscopic gates at the epidermal plant surface that can be opened or closed in response to environmental and endogenous signals. Stomatal movement depends largely on the content of ABA, considered the key hormone that closes stomata, although other hormones also participate in governing stomata responses (Wei et al. 2021). Under a subset of biotic stresses, ABA levels and stomata behavior play an essential plant defense role by hindering adversary access to the leaf interior (Lim et al. 2015; Melotto et al. 2017). In the plant–mite context, stomata are target sites where mites insert their stylets to access nutrient-rich mesophyll cells. Once the plant perceives the mites, a signal transduction cascade activates the synthesis of JA and SA, besides other chemical responses, to generate defenses (Zhurov et al. 2014; Santamaría et al. 2019). Our results confirmed that mite infestation induced JA and SA, demonstrated the timeline of this response, and revealed a mite-induced increase of ABA. Previous studies have established connections between stomatal movement, light response, circadian clock pathways, and pathways influenced by light changes, such as ROS (Rossel et al. 2002; Tallman 2004). We found that *T. urticae* caused less cell damage by feeding under dark conditions when stomata were closed. Concomitantly to stomatal closure, ABA accumulates in response to herbivory during the day. Additionally, the temporal pattern of the phenotypic changes in stomatal behavior in response to mite infestation during the day matched the ABA accumulation more than the JA or SA content. Together, these data pointed to ABA accumulation and stomatal closure as a plant response to herbivory acting to limit leaf cell damage associated with mite infestation.

Stomatal closure has been previously described as a plant response to other phytophagous species (Sances et al. 1979; Pincebourde and Casas 2006; Schmidt et al. 2009), though whether closure constituted a defense mechanism or contributed to the infestation remained unclear. DNA microarray experiments of Arabidopsis infiltrated with *M. persicae* saliva allowed the identification of a number of aphid saliva upregulated genes that also responded to ABA treatment (Hillwig et al. 2016). Aphid infestation induced ABA production in Arabidopsis; however, aphids preferred and performed better on WT plants than on ABA-deficient mutants, suggesting that ABA might not be a plant defense response. These authors also demonstrated that ABA increased the synthesis of some glucosinolates with defensive properties. We demonstrated a positive role for ABA in the plant defense to the mite *T. urticae* since mutant lines in ABA biosynthesis and catabolism showed higher and lower leaf damage, respectively, than Col-0 infested plants. These data directly corresponded to the higher and lower number of eggs accumulated in these mutants. Thus, mite infestation results in ABA accumulation as a plant defense response that closes stomata, reduces mite feeding ability, lessens leaf damage, and lowers mite fecundity rates.

Which mite-produced signal is detected and how this promotes protective ABA accumulation remains an open question. Plant damage, mite behavior, feeding vibrations, and microbes associated with the herbivore may elicit defense responses (Santamaria et al. 2020), and wounding has previously been shown to induce ABA accumulation (Zhang et al. 2021). Salivary proteins, feces, egg silks, and molts may provide HAMPs detected by plant pattern recognition receptors (PRRs). Of the characterized HAMPs, 2 proteins, Tetranin1 (Tet1) and Tetranin2 (Tet2), are candidates expressed in the salivary glands of *T. urticae* (Iida et al. 2019). Tet1 and Tet2 infiltration protects multiple plant species against herbivory, activating ROS and calcium responses and, in *Phaseolus vulgaris*, accumulation of several plant hormones (Iida et al. 2019).

Several possible biochemical mechanisms could integrate defense signaling into the ABA accumulation we observe during *T. urticae* infestation. De novo biosynthesis or deconjugation of the ABA glucosyl ester (ABA-GE) into bioactive ABA could increase the bioactive ABA pool, as could decreased catabolism or ABA repartitioning via mite-induced transporter dynamics. Our previous transcriptomics study of a mite infestation time series demonstrated early induction of several ABA-related enzymes and transporters (Santamaria et al. 2021), including 2 biosynthetic 9-CIS-EPOXYCAROTENOID DIOXYGENASE enzymes (*NCED5* at 1 h and NCED3 at 3 h; Tan et al. 2003) and an endoplasmic reticulum (ER)-localized ABA-GE deconjugation BETA-GLUCOSIDASE enzyme (*BGLU18* at 1, 3, and 24 h; Lee et al. 2006). Wound-induced leaf ER bodies containing BGLU18 have previously been shown to be critical for antiherbivory compound production, suggesting a possible dual role in mite defenses (Nakazaki et al. 2019). Key ABA catabolic enzymes, CYTOCHROME P450 707A and CYP707A1-A3, were induced by herbivory at all time points, suggesting that reduced catabolism is unlikely to drive ABA defense. Rather, as CYP707A enzymes are induced by ABA in the absence of water stress (Kushiro et al. 2004), their induction is consistent with negative feedback on ABA accumulation. Several ABA transporters also show a marked increase in expression during mite infestation at 1 or more time points. These include DETOXIFICATION EFFLUX CARRIER 50 (DTX50) and ABCG25, efflux carriers expressed in the vasculature (Kuromori et al. 2010; Zhang et al. 2014), and importers ABCG40 and ABCG18 that are expressed in the guard cells and mesophyll, respectively (Kang et al. 2010). Together, these results indicate that biosynthesis, deconjugation, and repartitioning from veins to other leaf cells could all contribute to the cellular ABA dynamics we observe in mite defense.

Vos et al. (2013) also reported that ABA is a crucial regulator of induced resistance against *P. rapae* by activating primed JA-regulated defense responses in Arabidopsis. Additionally, ABA has been associated with redox homeostasis (Li et al. 2022) and ROS accumulation, which is also essential in plant defense against mites (Santamaría et al. 2017; Arnaiz et al. 2021). Our results showed increased levels of JA signaling and H_2_O_2_ production in ABA-deficient mutants after mite infestation, where leaf damage was increased. Based on the current observations, it can be concluded that ABA signaling, independent of positive crosstalk with ROS and JA signaling, plays a critical role as a defense determinant against mite infestation. It remains to be seen how calcium signaling, which is often associated with ROS production (Takahashi et al. 2011), stress responses (Gilroy et al. 2014), and ABA (Edel and Kudla 2016) might interact with ABA-induced stomatal defense during *T. urticae* infestation.

To probe the regulation and function of ABA accumulation, we used nlsABACUS2, a FRET-based biosensor for ABA, as a tool to quantify in vivo ABA at high spatio-temporal resolution in response to mite infestation. Elevated levels of ABA were broadly detected in the nuclei of a range of leaf cell types, with slightly stronger responses in stomata and vascular tissues. These ABA dynamics are consistent with an ABA regulation model in which the leaf vasculature is the key site of biosynthesis triggered by mite infestation, with ABA subsequently transported to guard cells for stomatal closure, similar to that proposed for ABA during water stress by Kuromori et al. (2018). However, we also show induction of ABA in all leaf cells involved in feeding by mites, including pavement cells and the mesophyll cells on which they feed.

Given the pronounced stomatal closure observed during infestation, an additional significant aspect of this study focused on clarifying the impact of stomatal status versus ABA signaling more generally. This was achieved by administering exogenous ABA or FC to induce or repress stomatal closure. When pretreated with ABA and subsequently infested with mites, plants were protected without sacrificing their growth. Pretreatment with FC increased leaf damage, consistent with a protective role for stomatal closure. In an ABA + FC treatment, mite damage correlated with stomatal aperture rather than ABA levels, providing further support for the role of stomata in ABA-mediated mite defense. The closure of stomata likely hindered mite feeding through the natural openings on the leaf, compelling them to utilize their stylets for penetrating closed stomata or between the epidermal pavement cells. This mode of stylet penetration could trigger earlier damage and possibly defense signaling and likely reduces feeding success since mites try to avoid epidermal cell damage (Bensoussan et al. 2016), and we observed ABA to reduce overall leaf damage and defense signaling at 24 h infestation.

We also investigated the importance of leaf stomatal density upon infestation based on the hypothesis that a higher number of stomata could facilitate mite feeding. In mutants with more and fewer stomata, we observed lower and higher leaf temperatures that increased with mite feeding, indicating that mite-induced stomatal closure remained functional in leaves with altered stomatal density. Indeed, stomatal density was tightly correlated with mite-induced leaf damage, suggesting that the number of stomata, in conjunction with their aperture, is a highly relevant trait for plant resistance to mite infestation.

This study provides valuable insights into the important role of stomata and its regulation mediated by ABA in the Arabidopsis defense against *T. urticae*. We demonstrate that ABA, possibly of vascular origin, accumulates in the guard cells of the stomata as a result of mite feeding and triggers stomatal closure as a structural defense response. We also show that stomatal aperture and abundance play a crucial role in determining the success of an infestation. As both traits are regulated by ABA, the level of this phytohormone, which also integrates myriad other biotic and abiotic stress cues, is an important determinant of mite infestation that could be considered in breeding programs for pest control.

## Materials and methods

### Plant material and growth conditions

Arabidopsis (*A. thaliana*, ecotype Col-0), from Nottingham Arabidopsis Seed Collection (NASC; http://arabidopsis.info/BasicForm/), was used in all experiments as WT. Plants expressing nuclear-localized ABA FRET biosensors (nlsABACUS2-400n; Rowe et al. 2023) were used to quantify cellular ABA accumulation during mite feeding assays. *aba2-1* (González-Guzmán et al. 2002), *cyp707a1 cyp707a3* s (Okamoto et al. 2009), *pyr1pyl1pyl2pyl4pyl5pyl8* (hereafter *112458*; Gonzalez-Guzman et al. 2012), *ost1-3* (SALK_008068), and *epf1epf2* mutant lines and EPF2OE (Hara et al. 2009; Hunt et al. 2010) genotypes were used for ABA, stomata, and mite feeding assays. All plants were grown in a mixture of peat moss and vermiculite (2:1). Sterilized seeds were stratified in the dark at 4°C for 5 d, as in Santamaría et al. (2019). Plants were then grown in growth chambers (Sanyo MLR-351-H) under controlled conditions (23°C ± 1°C, >70% relative humidity, and a 16-h:8-h light:dark photoperiod). The growth chamber includes a light control program to mimic sunrise and sunset conditions.

### Spider mite growth, maintenance, and plant infestation

*T. urticae* London strain (Acari: Tetranychidae) population, provided by Dr. Miodrag Grbic (UWO, Canada), was reared on *P. vulgaris* (beans) and maintained in growth chambers (Sanyo MLR-351-H, Sanyo, Japan) at 25°C ± 1°C, >70% relative humidity, and a 16-h:8-h (light:dark) photoperiod. Mite infestation was performed in 3-wk-old Arabidopsis rosettes or detached leaves collected from the corresponding Arabidopsis genotypes with 20 mites/plant or 10 mites/leaf, adapting the infestation time according to the experiment. For leaf infestation assays, leaf number 5 or 6 was selected (Merchant and Pajerowska-Mukhtar 2015), placed on a one-half-strength MS (Duchefa Biochemie) medium plates, infested, and covered but ventilated with a relative humidity of 60% to 80%.

### Hormone analysis

Frozen plant material (ca. 50 mg) was used for the extractions. Plant samples were spiked with 25 *µ*L of an internal standard mixture (containing ABA-*d_6_*, DHJA, and C^13^-SA) to correct for analyte losses (De Ollas et al. 2021). Extraction was carried out in ultrapure water in a ball mill at room temperature using 2-mm glass beads. Homogenates were centrifuged at 10,000 rpm for 10 min at 4°C, and supernatants recovered. The resulting solutions were partitioned twice against an equal volume of diethyl ether after adjusting pH to 3.0 with a 30% acetic acid solution. The combined organic layers were evaporated under vacuum in a centrifuge concentrator (Jouan, Sant Germaine Cedex, France), and the dry residues were reconstituted in a 10% (w/v) aqueous methanol solution. Prior to injection, extracts were filtered through 0.20-*µ*m PTFE syringe membrane filters, and filtrates were recovered in chromatography amber glass vials. Samples were analyzed by tandem LC/MS in an Acquity SDS UPLC system (Waters Corp., United States) coupled to a TQS triple quadrupole mass spectrometer (Micromass Ltd., United Kingdom) through an electrospray ionization source. Separations were carried out on a C18 column (Luna Omega Polar C18, 50 × 2.1 mm, 1.6 *µ*m particle size, Phenomenex, United States) using a linear gradient of ultrapure acetonitrile and water, both supplemented with formic acid to a 0.1% (v/v) concentration, at a constant flow rate of 0.3 mL/min. During analyses, column temperature was maintained at 40°C and samples at 10°C to slow degradation. Plant hormones were detected in negative electrospray mode following their specific precursor-to-product ion transitions and quantitated using an external calibration curve with standards of known amount.

### Stomata leaf impression and aperture quantification

Stomata leaf impressions were made using detached leaves, with and without treatment and mite infestation (after a previous mite removal). Leaves were pressed on fast-setting dental resin AquasilUltra+ (Dentsply Sirona), which was allowed to set; then, the leaf material was removed. Transparent nail polish was spread onto the resin pieces (Wang et al. 2006). After they had thoroughly dried, nail varnish impressions were peeled using Sellotape and affixed to a glass microscope slide. These slides were then imaged on a Leica DM1000 LED with an ICC50 W camera. Stomatal density and aperture were quantified (Doheny-adams et al. 2012) with Fiji ImageJ software (Schindelin et al. 2012).

### Cell death quantification

Cell death quantification was performed by trypan blue staining after 16 h of infestation in light and dark conditions. Leaf disks were boiled in trypan blue solution, followed by a clarification process with 2.5 g/mL chloral hydrate (Sigma) solution (Sanchez-Vallet et al. 2010). Disks were placed onto glass slides in 50% (v/v) glycerol and observed under an epifluorescence stereoscope using UV filters. Pixel quantification was performed using Adobe Photoshop (Luna et al. 2012).

### Exogenous plant pretreatments

The stock solutions for hormone/FC treatments were dissolved in 96% ethanol and diluted in distilled water to make pretreatment solutions. Mock solutions and pretreatment solutions were matched, so plants received 0.19% ethanol. Exogenous treatments were applied to the aerial part of the whole plant by spraying with 1 mM SA, 1 mM JA, 10 *µ*M ABA, or 1 *µ*M FC (all from Sigma) unless stated in the figure. After 3 h of treatment, mite infestation or stomatal leaf impressions were performed. The mock treatment involved the same volume of ethanol as the pretreatment.

### Confocal microscopy and image processing

An inverted SP8 confocal microscope (Leica) was used for biosensor imaging assays in detached leaves. All images were acquired as Z-stacks in 16-bit mode, with a 10× dry objective. Samples were mounted in one-fourth MS, pH 5.7. Typical settings were as follows: sequential scanning was performed with excitation lasers and HYD detectors; 442 nm excitation 3% to 10% was used with HYD1 460 to 500 nm, 100 gain, for a first acquisition to detect the donor T7edCerulean fluorescence (donor excitation, acceptor emission or DxAm). Second, 442 nm excitation 3% to 10% was used with HYD2 525 to 560 nm, 100 gain, to acquire energy transfer fluorescence (donor excitation, donor emission or DxDm). Next, 514 nm excitation 5% to 10% was used with HYD2 525 to 560 nm, 100 gain, for a third acquisition to detect the edCitrineT7 acceptor protein fluorescence (acceptor excitation, acceptor emission or AxAm). The scan speed was set at 400; line averaging was 2 to 4, and bidirectional X was on.

### FRET cell and tissue classification

The image processing for fluorescence emission ratio (DxAm/DxDm) quantification was carried out using the “FRETNATOR” tool reported by Rowe et al. (2022, 2023) (Supplementary Fig. S5B). The AxAm images were used to segment the nuclei. The specific cell type emission ratio classification was done using the FretCellType extension plugin. The extension was developed using Apache Groovy and reads each series of acquisitions (Leica file format [.lif]). FretCellType uses the “FRETENATOR_Segment_and_ratio” output, specifically the “threshold image output” and “The label map,” to classify the nuclei based on the ROI shape. Cell types were defined as vascular bundle, bundle sheath, spongy mesophyll, pavement, and stomata cells. The plugin provides 2 tables (.csv) for each image: the “Result Table” and the “Summary Table.” The “Result Table” displays the number of pixels, the coordinates, and the emission ratio value for each nucleus. Supplementary Fig. S2B provides information about the nuclei classification shape validation.

### Plant damage and mite performance assessment

Chlorotic damage in whole plants was quantified 4 d after mite infestation, according to Ojeda-Martinez et al. (2020), using 9 biological replicates from independent rosettes for each genotype. Mite fecundity was determined using entire leaves detached from different Arabidopsis genotypes. Each leaf was infested with 10 synchronized females, and the number of eggs was counted after 36 h (Santamaría et al. 2019).

### Nucleic acid analysis

Total RNA was extracted from Arabidopsis rosettes by the phenol/chloroform method and precipitated with 8 M LiCl as described (Oñate-Sánchez and Vicente-Carbajosa 2008). Complementary DNAs (cDNAs) were synthesized from 2 *μ*g of RNA using the Revert Aid H Minus First Strand cDNA Synthesis Kit (Fermentas). RT-qPCR was performed using LightCycler 480 SYBR Green I Master (Roche), a SYBR Green Detection System (Roche), and the LightCycler 480 Software release 1.5.0 SP4 (Roche). mRNA quantification was expressed as relative expression levels (2^−dCt^) or as fold change (2^−ddCt^; Livak and Schmittgen 2001). Arabidopsis ubiquitin 21 was used as a housekeeping control gene and PR1 (Pathogenesis-Related protein-1) and *MYC2* (MYC2 transcription factor) as marker genes of SA and JA hormone signaling, respectively. Primer sequences are stated in Supplementary Table S4.

### Hydrogen peroxide determination

The accumulation of H_2_O_2_ was visualized in leaf disks after 24 h of mite infestation, using 3,3-diaminobenzidine tetrachloride hydrate (DAB, Sigma) as a substrate, according to De Ilarduya et al. (2003). Disks were placed onto glass slides in 50% (v/v) glycerol and observed under a stereoscope. Pixel quantification was performed with Fiji ImageJ software (Schindelin et al. 2012).

### Thermal imaging determination

Thermal images were obtained using a FLIR infrared camera (FLIR-T600) equipped with a 16° lens 24 h after whole plant infestation. To avoid changes in leaf temperature due to environmental conditions, experiments were conducted inside a walk-in growth chamber set at 25°C, 60 ± 10% relative humidity, and light intensity of 105 *μ*mol/m^2^/s. The camera was vertically mounted at approximately 20 cm above the setup. Images were saved as 8-bit TIFF files, and pictures were analyzed using ImageJ (Fiji), in which all pictures were set at a constant range of temperature based on pictures of Col-0 control conditions; the emissivity of the samples was 0.925.

### Statistical analysis

Statistical analyses were done using GraphPad Prism v9.4.1. The normality and homoscedasticity of the data were previously analyzed to apply the proper analysis. Student’s *t* test was used for individual analysis. One-way ANOVA followed by Tukey's multiple comparisons test was used to compare multiple data sets. Two-way ANOVA was performed in the experiments in which row data (R) and treatment with column data (C) were simultaneously analyzed, and Tukey's multiple comparisons test was used when the interaction (R × C) was significant. The correlation between damage and stomata density was analyzed by applying Pearson product moment correlation test. Statistical tests applied are listed in Supplementary Table S5.

### Accession numbers

Sequence data from this article can be found in the GenBank/EMBL data libraries under accession numbers AT2G14610 (PR1), AT1G32640 (MYC2), AT1G52340 (ABA2), AT4G19230 (CYP707A1), AT5G45340 (CYP707A3), AT4G33950 (OST1), AT4G17870 (PYR1), AT5G46790 (PYL1), AT2G26040 (PYL2), AT2G38310 (PYL4), AT5G05440 (PYL5), AT5G53160 (PYL8), AT2G20875 (EPF1), and AT1G34245 (EPF29).

## Supplementary Material

kiae215_Supplementary_Data

## Data Availability

FretCellType image analysis tool is available at https://github.com/acayuelalopez/FretCellType.

## Supplementary data

The following materials are available in the online version of this article.

**Supplementary Figure S1.** Quantification of nuclei number in different cell type groups of nlsABACUS2-400n plants after mite infestation in detached leaves.

**Supplementary Figure S2**. FRET image processing.

**Supplementary Figure S3.** Expression levels of PR1 and MYC2 genes in the 5 Arabidopsis genotypes.

**Supplementary Figure S4.** Redox status in the 5 Arabidopsis genotypes.

**Supplementary Figure S5.** Effects of different ratios of FC/ABA on stomata aperture in Arabidopsis Col-0 plants.

**Supplementary Table S1.** Statistical comparisons in Fig. 1A.

**Supplementary Table S2.** Effect size between SA, JA, and ABA treatments in Arabidopsis plants while measuring stomata aperture.

**Supplementary Table S3.** Leaf temperatures in Arabidopsis mutants of different stomatal densities after mite infestation.

**Supplementary Table S4.** Oligonucleotide sequences.

**Supplementary Table S5.** Statistical tests applied in the figures.

## Dive Curated Terms

The following phenotypic, genotypic, and functional terms are of significance to the work described in this paper:

OST1 Gramene: AT4G33950
OST1 Araport: AT4G33950

ABA2 Gramene: AT1G52340
ABA2 Araport: AT1G52340

EPF1 Gramene: AT2G20875
EPF1 Araport: AT2G20875

HYD1 Gramene: AT1G20050
HYD1 Araport: AT1G20050

NCED3 Gramene: AT3G14440
NCED3 Araport: AT3G14440

BGLU18 Gramene: AT1G52400
BGLU18 Araport: AT1G52400

NCED5 Gramene: At1g30100
NCED5 Araport: At1g30100

MYC2 Gramene: AT1G32640
MYC2 Araport: AT1G32640

PR1 Gramene: AT2G14610
PR1 Araport: AT2G14610

EPF2 Gramene: AT1G34245
EPF2 Araport: AT1G34245

pyl1 Gramene: AT5G46790
pyl1 Araport: AT5G46790

PYL2 Gramene: AT2G26040
PYL2 Araport: AT2G26040

PYL4 Gramene: At2g38310
PYL4 Araport: At2g38310

PYL8 Gramene: AT5G53160
PYL8 Araport: AT5G53160

ost1-3 Gramene: AT4G33950
ost1-3 Araport: AT4G33950

## References

[kiae215-B1] Aleman F , Yazaki J, Lee M, Takahashi Y, Kim AY, Li Z, Kinoshita T, Ecker JR, Schroeder JI. An ABA-increased interaction of the PYL6 ABA receptor with MYC2 transcription factor: a putative link of ABA and JA signaling. Sci Rep. 2016:6:28941. 10.1038/srep28941 27357749 PMC4928087

[kiae215-B2] Arnaiz A , Rosa-Diaz I, Romero-Puertas MC, Sandalio LM, Diaz I. Nitric oxide, an essential intermediate in the plant–herbivore interaction. Front Plant Sci. 2021:11:620086. 10.3389/fpls.2020.620086 33488661 PMC7819962

[kiae215-B3] Bari R , Jones JDG. Role of plant hormones in plant defence responses. Plant Mol Biol. 2009:69(4):473–488. 10.1007/s11103-008-9435-0 19083153

[kiae215-B4] Bensoussan N , Santamaria ME, Zhurov V, Diaz I, Grbić M, Grbić V. Plant-herbivore interaction: dissection of the cellular pattern of *Tetranychus urticae* feeding on the host plant. Front Plant Sci. 2016:7:1105. 10.3389/fpls.2016.01105 27512397 PMC4961969

[kiae215-B5] Bodenhausen N , Reymond P. Signaling pathways controlling induced resistance to insect herbivores in *Arabidopsis*. Mol Plant Microbe Interact. 2007:20(11):1406–1420. 10.1094/MPMI-20-11-1406 17977152

[kiae215-B6] Cazaux M , Navarro M, Bruinsma KA, Zhurov V, Negrave T, Van Leeuwen T, Grbic V, Grbic M. Application of two-spotted spider mite *Tetranychus urticae* for plant-pest interaction studies. J Vis Exp. 2014:4(89):e51738. 10.3791/51738 PMC4211727 25046103

[kiae215-B7] Chowdhury MR , Ahamed MS, Mas-ud MA, Islam H, Fatamatuzzohora M, Hossain MF, Billah M, Hossain MS, Matin MN. Stomatal development and genetic expression in *Arabidopsis thaliana* L. Heliyon 2021:7(8):e07889. 10.1016/j.heliyon.2021.e07889 34485750 PMC8408637

[kiae215-B8] Christmann A , Moes D, Himmelbach A, Yang Y, Tang Y, Grill E. Integration of abscisic acid signalling into plant responses. Plant Biol (Stuttg). 2006:8(3):314–325. 10.1055/s-2006-924120 16807823

[kiae215-B9] DeClerck RA , Steeves TA. Oviposition of the gall midge *Cystiphora sonchi* (Bremi) (Diptera: Cecidomyiidae) via the stomata of perennial sowthistle (*Sonchus arvensis* L.). Can Entomol. 1988:120(2):189–193. 10.4039/Ent120189-2

[kiae215-B10] De Ilarduya OM , Xie QG, Kaloshian I. Aphid-induced defense responses in Mi-1-mediated compatible and incompatible tomato interactions. Mol Plant Microbe Interact. 2003:16(8):699–708. 10.1094/MPMI.2003.16.8.699 12906114

[kiae215-B11] De Ollas C , González-Guzmán M, Pitarch Z, Matus JT, Candela H, Rambla JL, Granell A, Gómez-Cadenas A, Arbona V. Identification of ABA-mediated genetic and metabolic responses to soil flooding in tomato (*Solanum lycopersicum* L. Mill). Front Plant Sci. 2021:12:1–20. 10.3389/fpls.2021.613059 PMC7973378 33746996

[kiae215-B12] De Torres-Zabala M , Truman W, Bennett MH, Lafforgue G, Mansfield JW, Rodriguez Egea P, Bögre L, Grant M. *Pseudomonas syringae* pv. tomato hijacks the *Arabidopsis* abscisic acid signalling pathway to cause disease. EMBO J. 2007:26(5):1434–1443. 10.1038/sj.emboj.7601575 17304219 PMC1817624

[kiae215-B13] Dinh ST , Baldwin IT, Galis I. The HERBIVORE ELICITOR-REGULATED1 gene enhances abscisic acid levels and defenses against herbivores in *Nicotiana attenuata* plants. Plant Physiol. 2013:162(4):2106–2124. 10.1104/pp.113.221150 23784463 PMC3729786

[kiae215-B14] Doheny-adams T , Franks PJ, Doheny-adams T, Hunt L, Franks PJ, Beerling DJ, Gray JE. Genetic manipulation of stomatal density influences stomatal size, plant growth and tolerance to restricted water supply across a growth carbon dioxide gradient. Philos Trans R Soc Lond B Biol Sci. 2012:367(1588):547–555. 10.1098/rstb.2011.0272 22232766 PMC3248714

[kiae215-B15] Edel KH , Kudla J. Integration of calcium and ABA signaling. Curr Opin Plant Biol. 2016:33:83–91. 10.1016/j.pbi.2016.06.010 27366827

[kiae215-B16] Endo A , Sawada Y, Takahashi H, Okamoto M, Ikegami K, Koiwai H, Seo M, Toyomasu T, Mitsuhashi W, Shinozaki K, et al Drought induction of Arabidopsis 9-cis-epoxycarotenoid dioxygenase occurs in vascular parenchyma cells. Plant Physiol. 2008:147(4):1984–1993. 10.1104/pp.108.116632 18550687 PMC2492653

[kiae215-B17] Erb M , Reymond P. Molecular interactions between plants and insect herbivores. Annu Rev Plant Biol. 2019:70:527–557. 10.1146/annurev-arplant-050718-095910 30786233

[kiae215-B18] Garcia A , Santamaria ME, Diaz I, Martinez M. Disentangling transcriptional responses in plant defense against arthropod herbivores. Sci Rep. 2021:11(1):12996. 10.1038/s41598-021-92468-6 34155286 PMC8217245

[kiae215-B19] Gilroy S , Suzuki N, Miller G, Choi WG, Toyota M, Devireddy AR, Mittler R. A tidal wave of signals: calcium and ROS at the forefront of rapid systemic signaling. Trends Plant Sci. 2014:19(10):623–630. 10.1016/j.tplants.2014.06.013 25088679

[kiae215-B20] González-Guzmán M , Apostolova N, Bellés JM, Barrero JM, Piqueras P, Ponce MR, Micol JL, Serrano R, Rodríguez PL. The short-chain alcohol dehydrogenase ABA2 catalyzes the conversion of xanthoxin to abscisic aldehyde. Plant Cell. 2002:14(8):1833–1846. 10.1105/tpc.002477 12172025 PMC151468

[kiae215-B21] Gonzalez-Guzman M , Pizzio GA, Antoni R, Vera-Sirera F, Merilo E, Bassel GW, Fernández MA, Holdsworth MJ, Perez-Amador MA, Kollist H, et al Arabidopsis PYR/PYL/RCAR receptors play a major role in quantitative regulation of stomatal aperture and transcriptional response to abscisic acid. Plant Cell. 2012:24(6):2483–2496. 10.1105/tpc.112.098574 22739828 PMC3406898

[kiae215-B22] Grbić M , Van Leeuwen T, Clark RM, Rombauts S, Rouzé P, Grbić V, Osborne EJ, Dermauw W, Ngoc PCT, Ortego F, et al The genome of *Tetranychus urticae* reveals herbivorous pest adaptations. Nature 2011:479(7374):487–492. 10.1038/nature10640 22113690 PMC4856440

[kiae215-B23] Hara K , Yokoo T, Kajita R, Onishi T, Yahata S, Peterson KM, Torii KU, Kakimoto T. Epidermal cell density is autoregulated via a secretory peptide, EPIDERMAL PATTERNING FACTOR 2 in *Arabidopsis* leaves. Plant Cell Physiol. 2009:50(6):1019–1031. 10.1093/pcp/pcp068 19435754

[kiae215-B24] Hepworth C , Doheny-Adams T, Hunt L, Cameron DD, Gray JE. Manipulating stomatal density enhances drought tolerance without deleterious effect on nutrient uptake. New Phytol. 2015:208(2):336–341. 10.1111/nph.13598 26268722 PMC4973681

[kiae215-B25] Hillwig MS , Chiozza M, Casteel CL, Lau ST, Hohenstein J, Hernández E, Jander G, Macintosh GC. Abscisic acid deficiency increases defence responses against *Myzus persicae* in *Arabidopsis*. Mol Plant Pathol. 2016:17(2):225–235. 10.1111/mpp.12274 25943308 PMC6638517

[kiae215-B26] Holbein J , Franke RB, Marhavý P, Fujita S, Górecka M, Sobczak M, Geldner N, Schreiber L, Grundler FMW, Siddique S. Root endodermal barrier system contributes to defence against plant-parasitic cyst and root-knot nematodes. Plant J. 2019:100(2):221–236. 10.1111/tpj.14459 31322300

[kiae215-B27] Hsu P-K , Dubeaux G, Takahashi Y, Schroeder JI. Signaling mechanisms in abscisic acid-mediated stomatal closure. Plant J. 2021:105(2):307–321. 10.1111/tpj.15067 33145840 PMC7902384

[kiae215-B28] Hu Y , Ding Y, Cai B, Qin X, Wu J, Yuan M, Wan S, Zhao Y, Xin X-F. Bacterial effectors manipulate plant abscisic acid signaling for creation of an aqueous apoplast. Cell Host Microbe. 2022:30(4):518–529.e6. 10.1016/j.chom.2022.02.002 35247331

[kiae215-B29] Hunt L , Bailey KJ, Gray JE. The signalling peptide EPFL9 is a positive regulator of stomatal development. New Phytol. 2010:186(3):609–614. 10.1111/j.1469-8137.2010.03200.x 20149115

[kiae215-B30] Iida J , Desaki Y, Hata K, Uemura T, Yasuno A, Islam M, Maffei ME, Ozawa R, Nakajima T, Galis I, et al Tetranins: new putative spider mite elicitors of host plant defense. New Phytol. 2019:224(2):875–885. 10.1111/nph.15813 30903698

[kiae215-B31] Kang J , Hwang J-U, Lee M, Kim Y-Y, Assmann SM, Martinoia E, Lee Y. PDR-type ABC transporter mediates cellular uptake of the phytohormone abscisic acid. Proc Natl Acad Sci U S A. 2010:107(5):2355–2360. 10.1073/pnas.0909222107 20133880 PMC2836657

[kiae215-B32] Kuromori T , Miyaji T, Yabuuchi H, Shimizu H, Sugimoto E, Kamiya A, Moriyama Y, Shinozaki K. ABC transporter AtABCG25 is involved in abscisic acid transport and responses. Proc Natl Acad Sci U S A. 2010:107(5):2361–2366. 10.1073/pnas.0912516107 20133881 PMC2836683

[kiae215-B33] Kuromori T , Seo M, Shinozaki K. ABA transport and plant water stress responses. Trends Plant Sci. 2018:23(6):513–522. 10.1016/j.tplants.2018.04.001 29731225

[kiae215-B34] Kushiro T , Okamoto M, Nakabayashi K, Yamagishi K, Kitamura S, Asami T, Hirai N, Koshiba T, Kamiya Y, Nambara E. The *Arabidopsis* cytochrome P450 CYP707A encodes ABA 8′-hydroxylases: key enzymes in ABA catabolism. EMBO J. 2004:23(7):1647–1656. 10.1038/sj.emboj.7600121 15044947 PMC391058

[kiae215-B35] Lajeunesse G , Roussin-Léveillée C, Boutin S, Fortin É, Laforest-Lapointe I, Moffett P. Light prevents pathogen-induced aqueous microenvironments via potentiation of salicylic acid signaling. Nat Commun. 2023:14(1):713. 10.1038/s41467-023-36382-7 36759607 PMC9911384

[kiae215-B36] Lee KH , Piao HL, Kim H-Y, Choi SM, Jiang F, Hartung W, Hwang I, Kwak JM, Lee IJ, Hwang I. Activation of glucosidase via stress-induced polymerization rapidly increases active pools of abscisic acid. Cell 2006:126(6):1109–1120. 10.1016/j.cell.2006.07.034 16990135

[kiae215-B37] Li S , Liu S, Zhang Q, Cui M, Zhao M, Li N, Wang S, Wu R, Zhang L, Cao Y, et al The interaction of ABA and ROS in plant growth and stress resistances. Front Plant Sci. 2022:13:1050132. 10.3389/fpls.2022.1050132 36507454 PMC9729957

[kiae215-B38] Lim CW , Baek W, Jung J, Kim JH, Lee SC. Function of ABA in stomatal defense against biotic and drought stresses. Int J Mol Sci. 2015:16(12):15251–15270. 10.3390/ijms160715251 26154766 PMC4519898

[kiae215-B39] Lin P-A , Chen Y, Chaverra-Rodriguez D, Heu CC, Zainuddin NB, Sidhu JS, Peiffer M, Tan C-W, Helms A, Kim D, et al Silencing the alarm: an insect salivary enzyme closes plant stomata and inhibits volatile release. New Phytol. 2021:230(2):793–803. 10.1111/nph.17214 33459359 PMC8048682

[kiae215-B40] Livak KJ , Schmittgen TD. Analysis of relative gene expression data using real-time quantitative PCR and the 2-ΔΔCT method. Methods 2001:25(4):402–408. 10.1006/meth.2001.1262 11846609

[kiae215-B41] Luna E , Bruce TJA, Roberts MR, Flors V, Ton J. Next-generation systemic acquired resistance. Plant Physiol. 2012:158(2):844–853. 10.1104/pp.111.187468 22147520 PMC3271772

[kiae215-B42] Ma Y , Szostkiewicz I, Korte A, Moes D, Yang Y, Christmann A, Grill E. Regulators of PP2C phosphatase activity function as abscisic acid sensors. Science (1979). 2009:324(5930):1064–1068. 10.1126/science.1172408 19407143

[kiae215-B43] Mathen K , Radhakrishnan Nair CP, Gunasekharan M, Govindankutty MP, Solomon JJ. Stylet course of lace bug *Stephanitis typica* (Distant) in coconut leaf. Proc Anim Sci. 1988:97:539–544. 10.1007/BF03179555

[kiae215-B44] Melotto M , Underwood W, He SY. Role of stomata in plant innate immunity and foliar bacterial diseases. Annu Rev Phytopathol. 2008:46(1):101–122. 10.1146/annurev.phyto.121107.104959 18422426 PMC2613263

[kiae215-B45] Melotto M , Zhang L, Oblessuc PR, He SY. Stomatal defense a decade later. Plant Physiol. 2017:174(2):561–571. 10.1104/pp.16.01853 28341769 PMC5462020

[kiae215-B46] Merchant AM , Pajerowska-Mukhtar KM. *Arabidopsis thaliana* dynamic phenotype plasticity in response to enviromental conditions. Int J Mod Bot. 2015:5(2):23–28. 10.5923/j.ijmb.20150502.01

[kiae215-B47] Migeon A , Dorkeld F. Spider Mites Web: a comprehensive database for the Tetranychidae; 2023 [accessed 2023 Jan 23]. https://www1.montpellier.inra.fr/CBGP/spmweb.

[kiae215-B48] Munemasa S , Hossain MA, Nakamura Y, Mori IC, Murata Y. The Arabidopsis calcium-dependent protein kinase, CPK6, functions as a positive regulator of methyl jasmonate signaling in guard cells. Plant Physiol. 2011:155(1):553–561. 10.1104/pp.110.162750 20978156 PMC3075756

[kiae215-B49] Mustilli A , Merlot S, Vavasseur A, Fenzi F, Giraudat J. Arabidopsis OST1 protein kinase mediates the regulation of stomatal aperture by abscisic acid and acts upstream of reactive oxygen species production. Plant Cell. 2002:14(12):3089–3099. 10.1105/tpc.007906 12468729 PMC151204

[kiae215-B50] Nakazaki A , Yamad K, Kunied T, Sugiyam R, Hirai MY, Tamura K, Hara-Nishimura I, Shimada T. Leaf endoplasmic reticulum bodies identified in Arabidopsis rosette leaves are involved in defense against herbivory. Plant Physiol. 2019:179(4):1515–1524. 10.1104/pp.18.00984 30696747 PMC6446793

[kiae215-B51] Neill S , Barros R, Bright J, Desikan R, Hancock J, Harrison J, Morris P, Ribeiro D, Wilson I. Nitric oxide, stomatal closure, and abiotic stress. J Exp Bot. 2008:59(2):165–176. 10.1093/jxb/erm293 18332225

[kiae215-B52] Ng LM , Melcher K, Teh BT, Xu HE. Abscisic acid perception and signaling: structural mechanisms and applications. Acta Pharmacol Sin. 2014:35(5):567–584. 10.1038/aps.2014.5 24786231 PMC4813750

[kiae215-B53] Ojeda-Martinez D , Martinez M, Diaz I, Santamaria ME. Saving time maintaining reliability: a new method for quantification of *Tetranychus urticae* damage in *Arabidopsis* whole rosettes. BMC Plant Biol. 2020:20(1):397. 10.1186/s12870-020-02584-0 32854637 PMC7450957

[kiae215-B54] Okamoto M , Tanaka Y, Abrams SR, Kamiya Y, Seki M, Nambara E. High humidity induces abscisic acid 8′-hydroxylase in stomata and vasculature to regulate local and systemic abscisic acid responses in Arabidopsis. Plant Physiol. 2009:149(2):825–834. 10.1104/pp.108.130823 19036833 PMC2633821

[kiae215-B55] Oñate-Sánchez L , Vicente-Carbajosa J. DNA-free RNA isolation protocols for *Arabidopsis thaliana*, including seeds and siliques. BMC Res Notes. 2008:1(1):93. 10.1186/1756-0500-1-93 18937828 PMC2613888

[kiae215-B56] Pincebourde S , Casas J. Multitrophic biophysical budgets: thermal ecology of an intimate herbivore insect-plant interaction. Ecol Monogr. 2006:76(2):175–194. 10.1890/0012-9615(2006)076[0175:MBBTEO]2.0.CO;2

[kiae215-B57] Rossel JB , Wilson IW, Pogson BJ. Global changes in gene expression in response to high light in Arabidopsis. Plant Physiol. 2002:130(3):1109–1120. 10.1104/pp.005595 12427978 PMC166632

[kiae215-B58] Rowe J , Grangé-Guermente M, Exposito-Rodriguez M, Wimalasekera R, Lenz MO, Shetty KN, Cutler SR, Jones AM. Next-generation ABACUS biosensors reveal cellular ABA dynamics driving root growth at low aerial humidity. Nat Plants. 2023:9(7):1103–1115. 10.1038/s41477-023-01447-4 37365314 PMC10356609

[kiae215-B59] Rowe JH , Rizza A, Jones AM. Quantifying phytohormones in vivo with FRETFörster resonance energy transfer (FRET) biosensors and the FRETENATOR analysis toolset. In: Duque P, Szakonyi D, editors. Environmental responses in plants: methods and protocols. New York (NY): Springer US; 2022. p. 239–253. 10.1007/978-1-0716-2297-1_17 35467212

[kiae215-B60] Sances FV , Wyman JA, Ting IP. Morphological responses of strawberry leaves to infestations of twospotted spider mite. J Econ Entomol. 1979:72(5):710–713. 10.1093/jee/72.5.710

[kiae215-B61] Sanchez-Vallet A , Ramos B, Bednarek P, López G, Piślewska-Bednarek M, Schulze-Lefert P, Molina A. Tryptophan-derived secondary metabolites in *Arabidopsis thaliana* confer non-host resistance to necrotrophic *Plectosphaerella cucumerina* fungi. Plant Journal. 2010:63(1):115–127. 10.1111/j.1365-313X.2010.04224.x 20408997

[kiae215-B62] Santamaría ME , Martinez M, Arnaiz A, Ortego F, Grbic V, Diaz I. MATI, a novel protein involved in the regulation of herbivore-associated signaling pathways. Front Plant Sci. 2017:8:975. 10.3389/fpls.2017.00975 28649257 PMC5466143

[kiae215-B63] Santamaría ME , Martínez M, Arnaiz A, Rioja C, Burow M, Grbic V, Díaza I. An Arabidopsis TIR-lectin two-domain protein confers defense properties against *Tetranychus urticae*. Plant Physiol. 2019:179(4):1298–1314. 10.1104/pp.18.00951 30765478 PMC6446783

[kiae215-B64] Santamaria M , Arnaiz A, Rosa-Diaz I, González-Melendi P, Romero-Hernandez G, Ojeda-Martinez DA, Garcia A, Contreras E, Martinez M, Diaz I. Plant defenses against *Tetranychus urticae*: mind the gaps. Plants 2020:9(4):464. 10.3390/plants9040464 32272602 PMC7238223

[kiae215-B65] Santamaria ME , Garcia A, Arnaiz A, Rosa-Diaz I, Romero-Hernandez G, Diaz I, Martinez M. Comparative transcriptomics reveals hidden issues in the plant response to arthropod herbivores. J Integr Plant Biol. 2021:63(2):312–326. 10.1111/jipb.13026 33085192 PMC7898633

[kiae215-B66] Schindelin J , Arganda-Carreras I, Frise E, Kaynig V, Longair M, Pietzsch T, Preibisch S, Rueden C, Saalfeld S, Schmid B, et al Fiji: an open-source platform for biological-image analysis. Nat Methods. 2012:9(7):676–682. 10.1038/nmeth.2019 22743772 PMC3855844

[kiae215-B67] Schmidt L , Schurr U, Röse USR. Local and systemic effects of two herbivores with different feeding mechanisms on primary metabolism of cotton leaves. Plant Cell Environ. 2009:32(7):893–903. 10.1111/j.1365-3040.2009.01969.x 19302172

[kiae215-B68] Stahl E , Hilfiker O, Reymond P. Plant–arthropod interactions: who is the winner? Plant J. 2018:93(4):703–728. 10.1111/tpj.13773 29160609

[kiae215-B69] Suzuki T , España MU, Nunes MA, Zhurov V, Dermauw W, Osakabe M, Van Leeuwen T, Grbic M, Grbic V. Protocols for the delivery of small molecules to the two-spotted spider mite, *Tetranychus urticae*. PLoS One 2017:12(7):e0180658. 10.1371/journal.pone.0180658 28686745 PMC5501582

[kiae215-B70] Takahashi F , Mizoguchi T, Yoshida R, Ichimura K, Shinozaki K. Calmodulin-dependent activation of MAP kinase for ROS homeostasis in *Arabidopsis*. Mol Cell. 2011:41(6):649–660. 10.1016/j.molcel.2011.02.029 21419340

[kiae215-B71] Tallman G . Are diurnal patterns of stomatal movement the result of alternating metabolism of endogenous guard cell ABA and accumulation of ABA delivered to the apoplast around guard cells by transpiration? J Exp Bot. 2004:55(405):1963–1976. 10.1093/jxb/erh212 15310824

[kiae215-B72] Tan B-C , Joseph LM, Deng W-T, Liu L, Li Q-B, Cline K, McCarty DR. Molecular characterization of the *Arabidopsis* 9-cis epoxycarotenoid dioxygenase gene family. Plant J. 2003:35(1):44–56. 10.1046/j.1365-313X.2003.01786.x 12834401

[kiae215-B73] Thaler JS , Bostock RM. Interactions between abscisic-acid-mediated responses and plant resistance to pathogens and insects. Ecology 2004:85(1):48–58. 10.1890/02-0710

[kiae215-B74] Ton J , Flors V, Mauch-Mani B. The multifaceted role of ABA in disease resistance. Trends Plant Sci. 2009:14(6):310–317. 10.1016/j.tplants.2009.03.006 19443266

[kiae215-B75] Vacante V . The handbook of mites of economic plants: identification, bio-ecology and control. Wallingford (UK): CABI International; 2016.

[kiae215-B76] Vos IA , Verhage A, Schuurink RC, Watt LG, Pieterse CMJ, Van Wees SCM. Onset of herbivore-induced resistance in systemic tissue primed for jasmonate-dependent defenses is activated by abscisic acid. Front Plant Sci. 2013:4:539. 10.3389/fpls.2013.00539 24416038 PMC3874679

[kiae215-B77] Wang X , Mao Z, Choi K, Park K. Significance of the leaf epidermis fingerprint for taxonomy of genus *Rhododendron*. J For Res (Harbin). 2006:17(3):171–176. 10.1007/s11676-006-0041-1

[kiae215-B78] War AR , Paulraj MG, Ahmad T, Buhroo AA, Hussain B, Ignacimuthu S, Sharma HC. Mechanisms of plant defense against insect herbivores. Plant Signal Behav. 2012:7(10):1306–1320. 10.4161/psb.21663 22895106 PMC3493419

[kiae215-B79] Wei H , Jing Y, Zhang L, Kong D. Phytohormones and their crosstalk in regulating stomatal development and patterning. J Exp Bot. 2021:72(7):2356–2370. 10.1093/jxb/erab034 33512461

[kiae215-B80] Zamora O , Schulze S, Azoulay-Shemer T, Parik H, Unt J, Brosché M, Schroeder JI, Yarmolinsky D, Kollist H. Jasmonic acid and salicylic acid play minor roles in stomatal regulation by CO2, abscisic acid, darkness, vapor pressure deficit and ozone. Plant J. 2021:108(1):134–150. 10.1111/tpj.15430 34289193 PMC8842987

[kiae215-B81] Zhang H , Zhu H, Pan Y, Yu Y, Luan S, Li L. A DTX/MATE-type transporter facilitates abscisic acid efflux and modulates ABA sensitivity and drought tolerance in *Arabidopsis*. Mol Plant. 2014:7(10):1522–1532. 10.1093/mp/ssu063 24851876

[kiae215-B82] Zhang C , Žukauskaitė A, Petřík I, Pěnčík A, Hönig M, Grúz J, Široká J, Novák O, Doležal K. In situ characterisation of phytohormones from wounded *Arabidopsis* leaves using desorption electrospray ionisation mass spectrometry imaging. Analyst 2021:146(8):2653–2663. 10.1039/D0AN02118K 33661255

[kiae215-B83] Zhurov V , Navarro M, Bruinsma KA, Arbona V, Santamaria ME, Cazaux M, Wybouw N, Osborne EJ, Ens C, Rioja C, et al Reciprocal responses in the interaction between Arabidopsis and the cell-content-feeding chelicerate herbivore spider mite. Plant Physiol. 2014:164(1):384–399. 10.1104/pp.113.231555 24285850 PMC3875816

