# Supplementary material for: Spider mite herbivory induces an ABA-driven stomatal defense
Source: Plant Physiol. 2024 Apr 26;195(4):2970–84. doi: 10.1093/plphys/kiae215 (PMC11288753; doi:10.1093/plphys/kiae215)
Supplement: kiae215_Supplementary_Data [file kiae215_supplementary_data.zip › kiae215_Supplementary_Data.pdf]

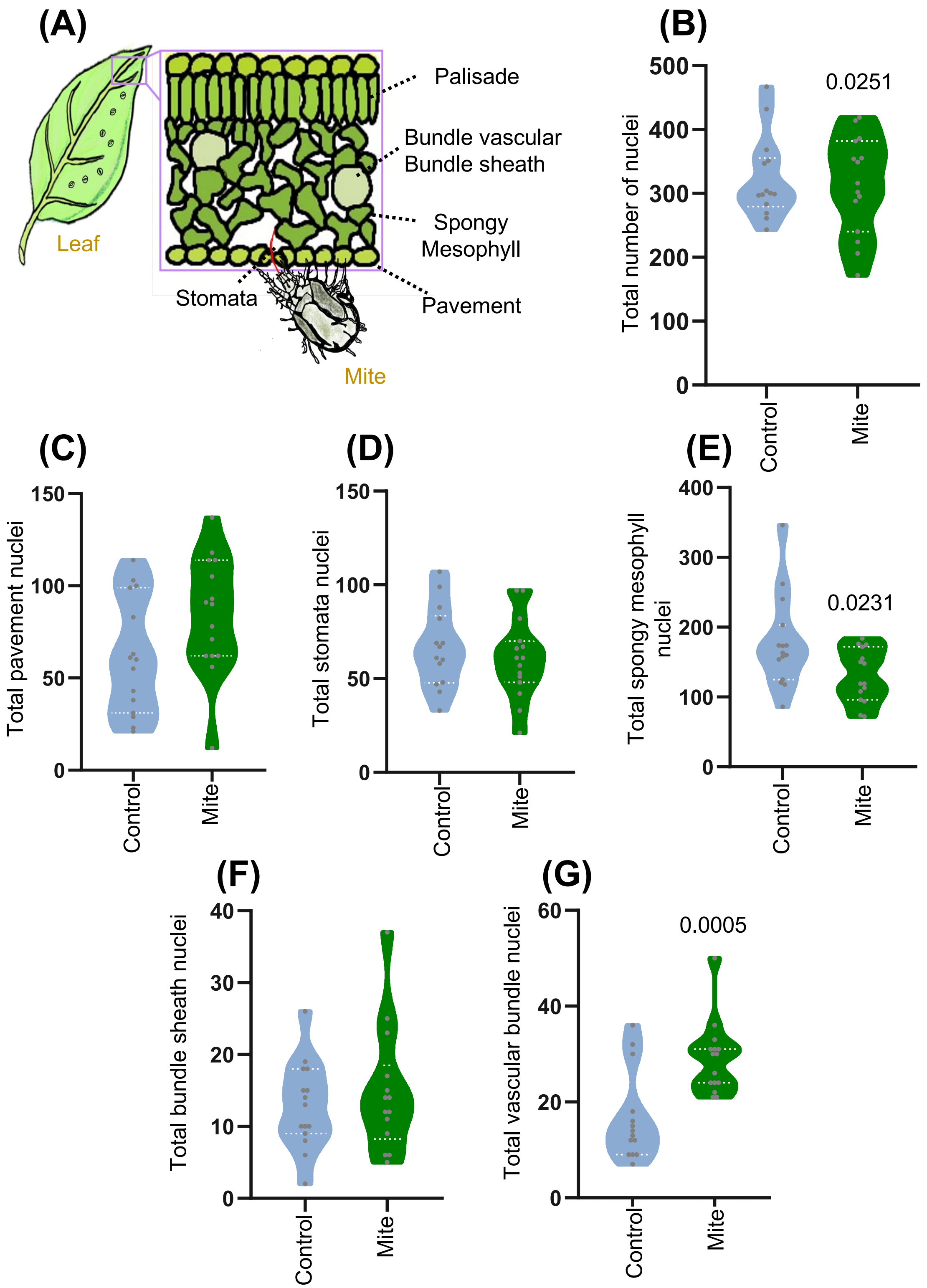

Supplementary Figure S1. Quantification of nuclei number in different cell type groups of nls-ABACUS2-400n plants after mite infestation in detached leaves. (A) Scheme of the leaf tissues and a mite feeding through stomata. (B) Total number detected nuclei in infested and control leaves. (C-G) Total number of nuclei in different cell types: Pavement (C), Stomata (D), Spongy mesophyll (E), Bundle sheath (F) and Vascular bundle (G), after 24 h of mite infestation. Numbers indicate significant differences between control and mite treatment. A Student T-test was used to assess differences due to mite treatments ($P<0.05$). Data are means nuclei from of 15 replicate leaves.

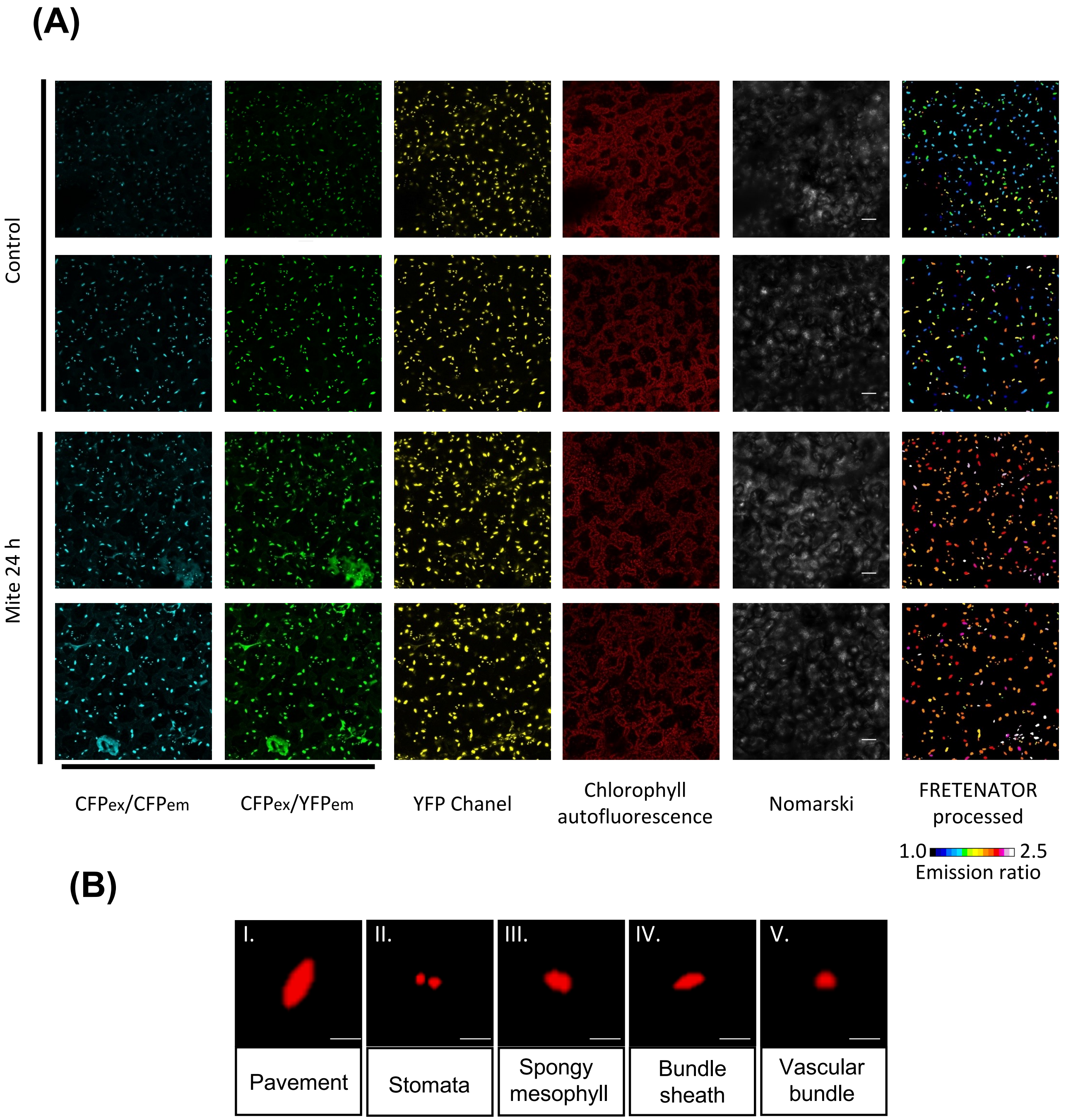

Supplementary Figure S2. Forster Resonance Energy Transfer (FRET)-image processing. (A) Z-projections of raw and processed confocal images after 24 h of mite infestation. For FRETENATOR processed images, black areas represent areas excluded from analysis during segmentation. Bars = 50µm (B) FretCellType nucleus determination based on their shape. I. Pavement nuclei. II. Stomata nuclei III. Spongy mesophyll nuclei, IV. Bundle sheath nuclei, V. Vascular bundle nuclei. In all the images black areas represent areas excluded from analysis during segmentation. Bars = 10µm

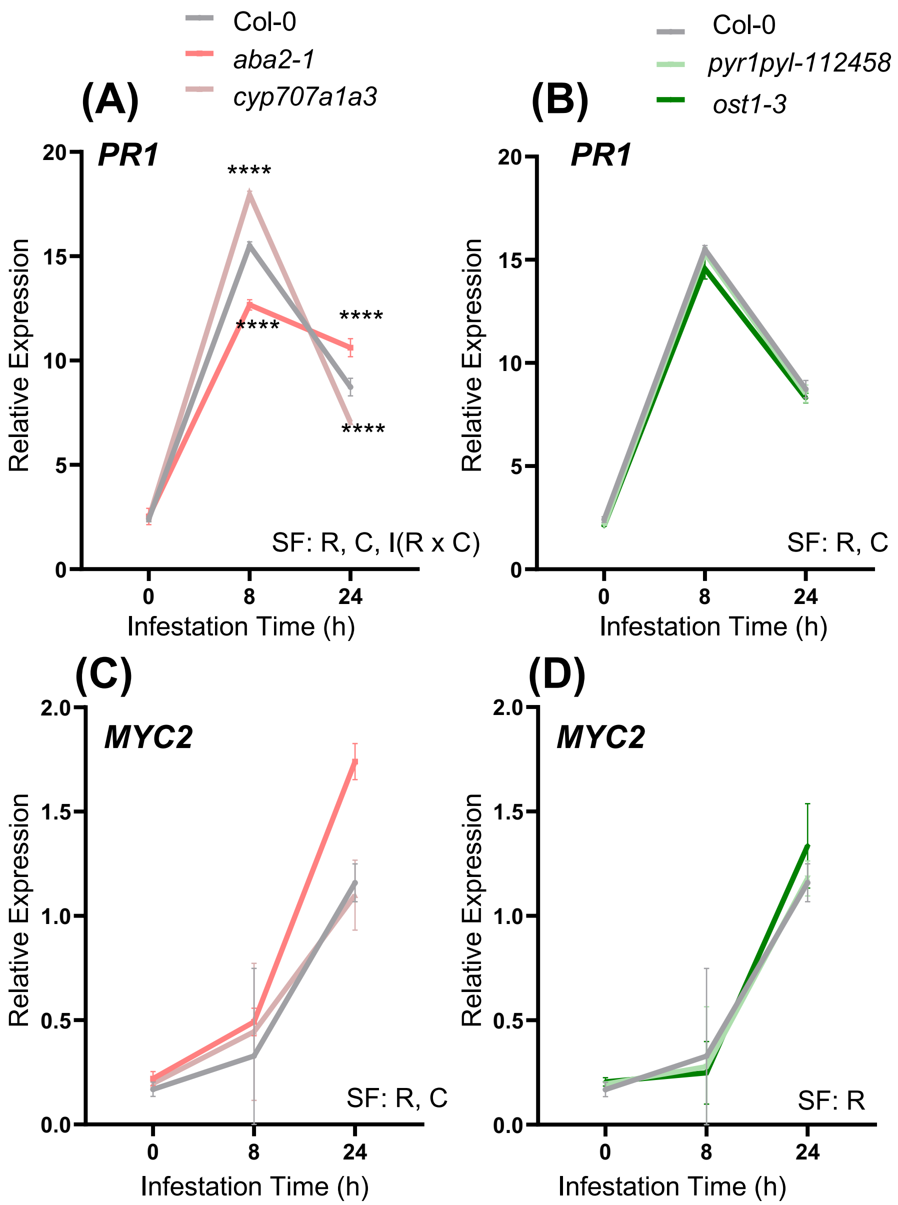

Supplementary Figure S3. Expression levels of *PR1* and *MYC2* genes in the five Arabidopsis genotypes. *PR1* gene (A, B) and *MYC2* gene (C, D). Gene expression levels were determined in *aba2-1*, *cyp707a1cyp707a3,* and in *pyr1pyl-112458, ost1-3* and Col-0 whole plants. Values indicated as relative expression. Significant factors (SF) indicate whether the two independent factors, R (infestation time) and C (genotype), and/or their interaction I (RxC) were statistically significant (Two-way ANOVA followed by Tukey's multiple comparison test, P<0.05). Asterisks indicate groups that are significantly different from Col-0 mock at that time point. Detailed ANOVA results are available in Supplemental Table S5. Data are means ± SE of 3 pools of 6 biological replicates.

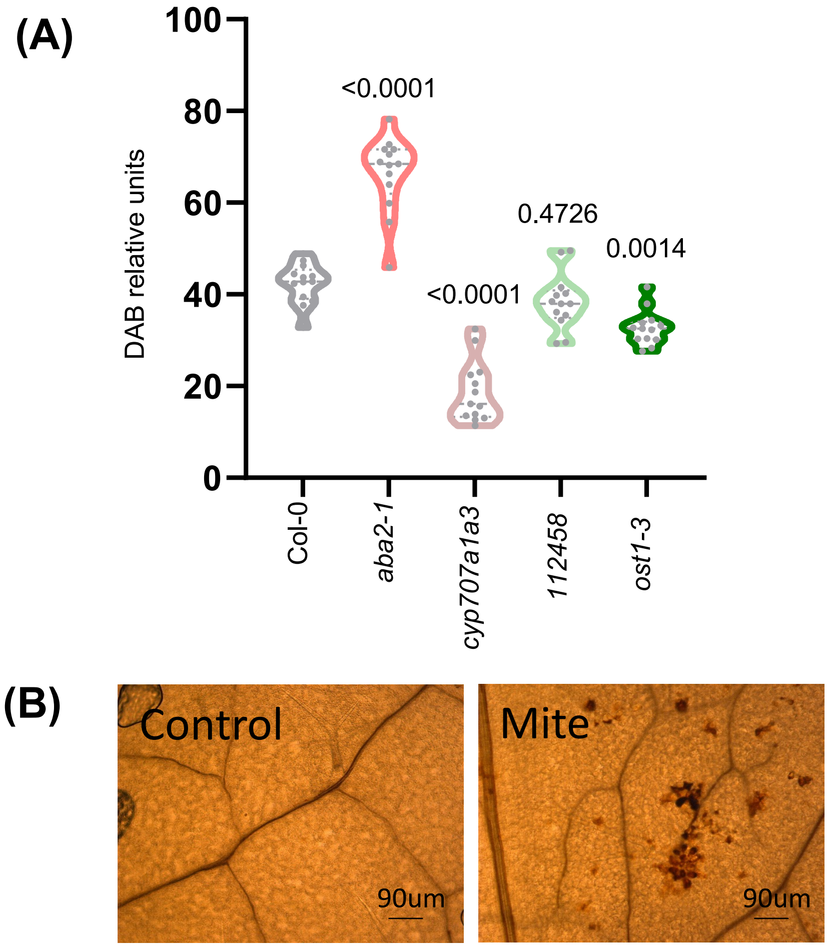

Supplementary Figure S4. Redox status in the five Arabidopsis genotypes. (A) Accumulation of H_2O_2 in leaf discs after 24 h of mite infestation, expressed as 3, 3'-diaminobenzidine (DAB) units. Numbers indicate p values when compared to Col-0 genotype. Data are means of 13 biological replicates. One way ANOVA followed by Tukey's multiple comparison test, $P<0.05$). Detailed ANOVA results are available in Supplemental Table S5. Data are means ± SE of 3 pools of 6 biological replicates. (B) Pictures showing the H_2O_2 deposits during *T. urticae* feeding.

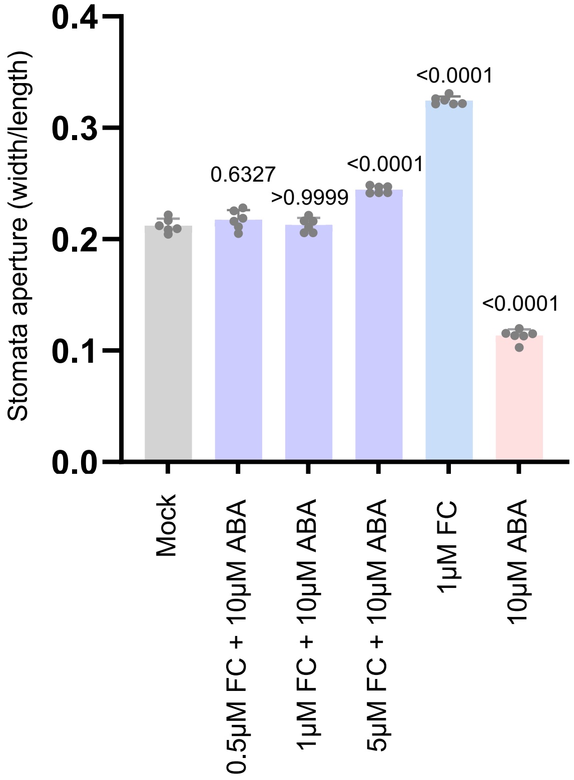

Supplementary Figure S5. Effects of different ratios of Fusicoccin: Abscisic Acid on stomata aperture in Arabidopsis Col-0 plants. Stomata aperture of Arabidopsis detached leaves pre-treated with 10 μM abscisic acid (ABA), 1 μM of fusicoccin (FC), or a combination of 10 μM ABA plus different concentrations (0.5, 1.0, and 5.0 μM) of FC. Numbers indicate p values compared to mock treatment**.** Detailed ANOVA results are available in Supplemental Table S5. Data are means ± SE of 3 pools of 6 biological replicates. Data are means of 6 biological replicate leaves (One-way ANOVA followed by Tukey's multiple comparison test, $P<0.05$).

Supplementary Table S1. Statistical comparisons in Figure 1A

Sidak's multiple comparisons test	Mean Diff,	95,00% CI of diff,	Significant	Summary	Adjusted P Value
3:Control vs. 3:Mite	0.004854	-0,001745 to 0,01145	No	ns	0.4515
3:Control vs. 8:Control	0.01539	0,008790 to 0,02199	Yes	****	<0,0001
3:Control vs. 8:Mite	0.03121	0,02461 to 0,03781	Yes	****	<0,0001
3:Control vs. 24:Control	-0.004325	-0,01092 to 0,002274	No	ns	0.6794
3:Control vs. 24:Mite	0.07091	0,06431 to 0,07751	Yes	****	<0,0001
3:Control vs. 30:Control	0.0121	0,005505 to 0,01870	Yes	****	<0,0001
3:Control vs. 30:Mite	0.0626	0,05600 to 0,06920	Yes	****	<0,0001
3:Mite vs. 8:Control	0.01054	0,003937 to 0,01713	Yes	****	<0,0001
3:Mite vs. 8:Mite	0.02636	0,01976 to 0,03296	Yes	****	<0,0001
3:Mite vs. 24:Control	-0.009179	-0,01578 to -0,002580	Yes	***	0.0005
3:Mite vs. 24:Mite	0.06605	0,05945 to 0,07265	Yes	****	<0,0001
3:Mite vs. 30:Control	0.00725	0,0006511 to 0,01385	Yes	*	0.0176
3:Mite vs. 30:Mite	0.05775	0,05115 to 0,06435	Yes	****	<0,0001
8:Control vs. 8:Mite	0.01582	0,009223 to 0,02242	Yes	****	<0,0001
8:Control vs. 24:Control	-0.01971	-0,02631 to -0,01312	Yes	****	<0,0001
8:Control vs. 24:Mite	0.05552	0,04892 to 0,06212	Yes	****	<0,0001
8:Control vs. 30:Control	-0.003286	-0,009885 to 0,003313	No	ns	0.9699
8:Control vs. 30:Mite	0.04721	0,04062 to 0,05381	Yes	****	<0,0001
8:Mite vs. 24:Control	-0.03554	-0,04213 to -0,02894	Yes	****	<0,0001
8:Mite vs. 24:Mite	0.0397	0,03310 to 0,04630	Yes	****	<0,0001
8:Mite vs. 30:Control	-0.01911	-0,02571 to -0,01251	Yes	****	<0,0001
8:Mite vs. 30:Mite	0.03139	0,02479 to 0,03799	Yes	****	<0,0001
24:Control vs. 24:Mite	0.07523	0,06863 to 0,08183	Yes	****	<0,0001
24:Control vs. 30:Control	0.01643	0,009830 to 0,02303	Yes	****	<0,0001

24:Control vs. 30:Mite	0.06693	0,06033 to 0,07353	Yes	****	<0,0001
24:Mite vs. 30:Control	-0.0588	-0,06540 to -0,05220	Yes	****	<0,0001
24:Mite vs. 30:Mite	-0.008304	-0,01490 to -0,001705	Yes	**	0.0027
30:Control vs. 30:Mite	0.0505	0,04390 to 0,05710	Yes	****	<0,0001

Supplementary Table S2. Effect size between SA, JA, and ABA treatments in Arabidopsis plants while measuring stomata aperture.

	SA	JA	ABA
Mean of Mock	0.25	0.25	0.25
Mean of Hormone	0.17	0.25	0.11
Difference between means (Hormone - Mock) ± SEM	-0.080 ± 0.003	-0.006 ± 0.002	-0.14 ± 0.002
95% confidence interval	-0.087 to -0.073	-0.012 to -0.0008	-0.1500 to -0.1387
R squared (eta squared)	0.909	0.088	0.980

Supplementary Table S3. Leaf temperatures in Arabidopsis mutants of different stomatal density after mite infestation. Data are mean ± SE**.**

Treatment	Mean (ºC)		
	**Col-0**	***epf1epf2***	***EPF2OE***
Control	19.2 ± 0.03	17.4 ± 0.03	20.5 ± 0.08
Mite	21.4 ± 0.09	20.4 ± 0.04	21.5 ± 0.03

Supplementary Table S4. Oligonucleotide sequences. Primer sequences used for RT-qPCR assay.

Gene	Primer name	Sequence 5'→3'	Purpose
At2g14610	PR1-Fw	TCAGTGAGACTCGGATGTGC	RT-qPCR
	PR1-Rv	CGTTCACATAATTCCCACGA	
At1g32640	MYC2-Fw	TCCGAGTCCGGTTCATTCT	
	MYC2-Rv	TCTCGGGAGAAAGTGTTATTGAA	

Supplementary Table S5. Statistical tests applied in the figures.

Figure	Panel	P-value T-student	Significant	Replicates
1	c	0.0021	Yes	15 Br
2	e	<0,0001	Yes	10 Br
	f	0.0257	Yes	
	g	<0,0001	Yes	
3	b	0,0100	Yes	8 Br
5	a.Mock	<0,0001	Yes	8 Br
	a.ABA	<0,0001	Yes	
	b.Mock	<0,0001	Yes	9 Br
	b.ABA	0.1326	No	
	c	0.0002	Yes	10 Br
	d.Control	0.1087	No	
	d.Mite	0.0097	Yes	
S1	b	0.0251	Yes	8 Br
	c	0.0577	No	
	d	0.447	No	
	e	0.0231	Yes	
	f	0.5085	No	
	g	0.005	Yes	
**Figure**	**Panel**	**P-value Pearson Product Moment**	**Pearson r**	
7	E	0.0569	0.996	
**Figure**	**Panel**	**P-value One Way ANOVA**	**Significant**	**Replicates**
4	a	<0,0001	Yes	10 Br
	b	<0,0001	Yes	
	c	<0,0001	Yes	6 Br
	d	0.2387	No	
6	c	<0,0001	Yes	9 Br
7	a	<0,0001	Yes	9 Br
	d	<0,0001	Yes	10 Br
S3	a	<0,0001	Yes	10 Br
S4		<0,0001	Yes	8 Br

Figure	Panel	P-value Two Way ANOVA			Replicates
		I	Row	Column	

1		Interaction	Timing	Treatment	
	a	<0,0001	<0,0001	<0,0001	10 Br
2		Interaction	Timing	Treatment	
	a	<0,0001	<0,0001	<0,0001	3 groups of 6 Br
	b	0.0472	0.0045	<0,0001	
	c	0.007	0.0035	0.0001	
	d	0.018	0.003	<0,0001	
3		Interaction	Treatment	Nucleus type	
	c	0.0317	<0,0001	0.0004	8 Br
6		Interaction	Treatment	Pre-treatment	
	b	<0,0001	<0,0001	0.0005	8 Br
7		Interaction	Treatment	Genotype	
	b	<0,0001	<0,0001	<0,0001	8 Br
	c	<0,0001	<0,0001	<0,0001	
S2		Interaction	Timing	Genotype	
	a	<0,0001	<0,0001	0.0026	3 groups of 6 Br
	b	0.2407	<0,0001	0.0035	
	c	0.0576	<0,0001	0.0172	
	d	0.8255	<0,0001	0.8517	

Br: Biological replicates details	
Timing	Infestation time (h)
Treatment	Control/Mite
Pre-treatment	Exogenous applications before mite infestation
Genotype	Arabidopsis mutant lines
