# Supplementary material for: Spider mite herbivory induces an ABA-driven stomatal defense
Source: Plant Physiol. 2024 Apr 26;195(4):2970–84. doi: 10.1093/plphys/kiae215 (PMC11288753; doi:10.1093/plphys/kiae215)
Supplement: kiae215_Supplementary_Data [file pp.01949.2023-s03.pdf › pp.01949.2023-s03.pdf]

Plant Physiology®

Spider mite herbivory induces an abscisic acid-driven stomatal defense

Irene Rosa-Diaz, James Rowe, Ana Cayuela-Lopez, Vicent Arbona, Isabel Diaz, and Alexander Jones

Review Timeline:

Submission Date:	29-Aug-2023
Editorial Decision:	27-Oct-2023
Resubmission Received:	08-Dec-2023
Editorial Decision:	08-Jan-2024
Revision Received:	26-Jan-2024
Accepted:	14-Mar-2024

25-Oct-2023

Dr. Alexander M Jones
Sainsbury Laboratory Cambridge University
Sainsbury Laboratory
Cambridge, Please select an option below CB2 1LR
United Kingdom

RE: Stomata, a vulnerability in the plant defence against phytophagous mites that ABA can overcome

Dear Dr. Alexander Jones:

Thank you for giving us the opportunity to review your manuscript for publication in the Plant Physiology. The reviewers have found the manuscript of interest, but have raised a number of concerns that will require your attention. As these concerns are of some gravity and will entail substantial changes to the manuscript, you are likely to need additional time to address them. I must therefore reject the manuscript in its present form. Nonetheless, if you are able to address the points raised in review, I would welcome your resubmission of a manuscript that incorporates your responses.

In particular, I direct your attention to the following comments for consideration in a resubmitted manuscript.

Please modify the title and the subtitle

Importantly, include control plant areas to compare with mite-infestation

Modify the discussion about the difference between light and dark conditions to take to into consideration all aspect of plant's responses

Please correct the statement of the difference between infested and non-infested plants in the number of viable nuclei

Clarify the M&M section according to the referee's comments

Correct Table S4, and add sample sizes to figure legends

Clarify the statistical analysis.

Mechanistic details on how mite infestation induces the ABA accumulation could be more extensively discussed

Please also answer all the others points raised by the reviewers.

Please note that any resubmission will be treated as a new entry to the system and is likely to go out for review.

If you do have any queries or concerns, you are welcome to contact me in the first instance.

Thank you again for allowing Plant Physiology to review your work. We look forward to hearing from you soon.

Sincerely,

Board of Editors, Plant Physiology

-- Reviewer comments:
Reviewer #1 (Comments for the Author):

The manuscript describes a role for ABA and stomata in spider mite infestation in Arabidopsis. The findings indicate that open stomata facilitate mite feeding, mites induce ABA production in plants, and stomatal closure and low stomatal numbers can protect plants from damage caused by spider mite infestation. Thus, the manuscript is of broad interest, contributing to fields of plant defence responses and stomatal biology. There are some issues that should be addressed by the authors to ensure that interpretations fully match the data as specified below.

Specific comments:
Line 169 - the subtitle "Stomata responses to T.urticae infestation are driven by hormone content" is not justified - while stomata close and hormones are induced after infestation, a causal relationship is not shown by the presented experiments in the respective section. The subtitle should be revised.

Lines 177-181 - the authors interpret higher cell death area in mite-infested plants under light, as opposed to dark conditions (Fig. 1C), as a result of stomatal openness or closure, respectively, that might affect mite feeding. However, as light conditions stimulate e.g. ROS production and cell death signalling, it is possible that the difference between light and dark conditions is not related with stomatal apertures, but e.g. different induction of cell death responses under light and dark conditions. Thus, I think the interpretation needs to be revised for this experiment.

Line 219-221 the authors state that there was no difference between infested and non-infested plants in the number of viable nuclei, but in Figure S1B, there is a statistically significant difference between groups. Please correct.

Figure 1A. It is not clear, what the asterisks and p-values show: the legend states that they show differences between light and dark conditions, but in the figure all data points seem to be sampled in the light. Please clarify.

Figure 2E-G, lines 539-542. Please elaborate in the methods section, what was used as mock treatment. It is unclear, how much ethanol was applied to plants and whether pH was taken into account. For example, the pH of 1 mM SA should be about 3 - this is rather acidic and thus it is unclear, if the observed stomatal closure was due to SA or due to acid treatment.

Figure 5D, E. It is difficult to conclude whether ABA-treatment increased growth under mite-infestation, because areas for control-treated plants are not shown. Please include control plant areas.

Figure 7A, E. Would be good to show also true stomatal density values per unit leaf area, at least as a supplemental figure. At present, it seems something has gone rather wrong with these numbers, as numbers that give log10 values of 10-60 are extremely large.

Table S4. Figure panel letters in the table do not match the figures. For example, there are only A, B C panels in Figure 1, but according to Table S4, there should also be D-G. Please correct.

General comments:
1) Overall comment on indicating statistical significance in figures - I think there is no reason to show both the p-value and asterisks. The p-value alone is sufficient and more informative than the asterisks, so I suggest to show only p-values. Further, if there is a specific p-value and not a comparison with a significance threshold, such as 0.0021 in Fig 1C, I suggest to state $p = 0.0021$, not $p < 0.0021$.

2) I also suggest to consider using Tukey post hoc test after two-way ANOVA instead of the Student-Newman-Keuls test, as the latter is more prone to type I errors and is usually not recommended when more than three groups' means are compared. The Methods section even says that Tukey test has been used, but this does not match the legends.

3) I suggest adding sample sizes to each figure legend.

4) I suggest to revise the tables with respect to the precision and significant digits of the presented numbers - for example, in Table S2, if the mean is presented with one digit after decimal point, presenting the measures of errors with three does not make sense. It is also questionable, whether 4 significant digits are justified for the precision of stomatal aperture measurements presented in Table S1.

5) Although several legends say that single factors' (e.g. infestation time and mite treatment; or mite treatment and nucleus type) significance is indicated by R and C, these are not shown in the figures, only interaction effects are shown. Showing only significant interaction is fine, but the legends are confusing, if presented this way.

6) Plant infestation: it is not clear, which experiments used whole plant and which used detached leaves, would be good to include this in figure legends.

Reviewer #2 (Comments for the Author):

In the Ms, authors explored Arabidopsis response to mite T. urticae infestation and established the role of ABA in stomatal closure to effectively hinders mite infestation. The study is intresting, moreover the role of ABA is already well established in stomata defence in water stress. This study provides an interesting relation between stomatal aperture and mite infestation. Introduction is nicely written with the updated information and gap areas.
Authors found that the accumulation of SA, JA, JA-Ile and ABA was increased after mite infestation, especially the ABA. Further, they found a correlation between ABA, stomatal closure and mite infestation by using numerous approaches including various

ABA related mutants analysis, exogenous application of ABA etc.
It seems to be a complete study, moreover, it would be more interesting if authors may include some mechanistic details.
How mite infestation induces the ABA accumulation should be included. Besides, authors analysed the ROS, but usually ROS and calcium signalling are related to each other, what is the status of Calcium in cell may also be checked.
Authors analysed the ABA biosynthesis and catabolism related mutants aba2-1 and cyp707a1cyp707a3, along with e 112458 ABA receptor sextuple mutant and ost1-3 SnRK2 kinase mutant . But, they have not studied any sensor elements. ABA usually functions downstream or slightly late responsive. It will be interesting if authors identify the sensor protein which first recognizes the mite infestation, and then induces the ABA biosynthesis and further downstream signalling.

The title also not seems to be appealing.

Response to Reviewers comments – Rosa-Diaz et al Plant Physiology

"Spider mite herbivory induces an abscisic acid-driven stomatal defense"

Dear Dr. Alexander Jones:

Thank you for giving us the opportunity to review your manuscript for publication in the Plant Physiology. The reviewers have found the manuscript of interest, but have raised a number of concerns that will require your attention. As these concerns are of some gravity and will entail substantial changes to the manuscript, you are likely to need additional time to address them. I must therefore reject the manuscript in its present form. Nonetheless, if you are able to address the points raised in review, I would welcome your resubmission of a manuscript that incorporates your responses.

In particular, I direct your attention to the following comments for consideration in a resubmitted manuscript.
Please modify the title and the subtitle
Importantly, include control plant areas to compare with mite-infestation
Modify the discussion about the difference between light and dark conditions to take to into consideration all aspect of plant's responses

Please correct the statement of the difference between infested and non-infested plants in the number of viable nuclei
Clarify the M&M section according to the referee's comments
Correct Table S4, and add sample sizes to figure legends
Clarify the statistical analysis.
Mechanistic details on how mite infestation induces the ABA accumulation could be more extensively discussed
Please also answer all the others points raised by the reviewers.
Please note that any resubmission will be treated as a new entry to the system and is likely to go out for review.

If you do have any queries or concerns, you are welcome to contact me in the first instance.
Thank you again for allowing Plant Physiology to review your work. We look forward to hearing from you soon.
Sincerely,
Board of Editors, Plant Phys

Reviewer comments:
____-
Reviewer #1 (Comments for the Author):

The manuscript describes a role for ABA and stomata in spider mite infestation in Arabidopsis. The findings indicate that open stomata facilitate mite feeding, mites induce ABA production in plants, and stomatal closure and low stomatal numbers can protect plants from damage caused by spider mite infestation. Thus, the manuscript is of broad interest, contributing to fields of plant defence responses and stomatal biology. There are some issues that should be addressed by the authors to ensure that interpretations fully match the data as specified below.

Specific comments:

Line 169 - the subtitle "Stomata responses to T. urticae infestation are driven by hormone content" is not justified - while stomata close and hormones are induced after infestation, a causal relationship is not shown by the presented experiments in the respective section. The subtitle should be revised.

Many thanks for your insightful feedback. We are grateful for the time and consideration you put into reviewing the work. Your input has been enlightening and is greatly appreciated. We have changed the subtitle to:

———*T. urticae* infestation induces stomata closure and modifies hormone content

Lines 177-181 - the authors interpret higher cell death area in mite-infested plants under light, as opposed to dark conditions (Fig. 1C), as a result of stomatal openness or closure, respectively, that might affect mite feeding. However, as light conditions stimulate e.g. ROS production and cell death signalling, it is possible that the difference between light and dark conditions is not related with stomatal apertures, but e.g. different induction of cell death responses under light and dark conditions. Thus, I think the interpretation needs to be revised for this experiment.

Thank you for this helpful critique, we have revised our comments on this topic in the results and discussion.

Line 219-221 the authors state that there was no difference between infested and non-infested plants in the number of viable nuclei, but in Figure S1B, there is a statistically significant difference between groups. Please correct.

Thank you, this was a mistake and the sentence has been corrected. (Line 217-218)

Figure 1A. It is not clear, what the asterisks and p-values show: the legend states that they show differences between light and dark conditions, but in the figure all data points seem to be sampled in the light. Please clarify.

We want to express our appreciation for your detailed review. To be clear, all sampling was performed in the light in this experiment. The figure, legend and accompanying text (Lines 166-172) have been modified and Table S1 has been added for clarity. Tables S1 and S5 display the complete statistical analysis of these samples and comparisons.

Figure 2E-G, lines 539-542. Please elaborate in the methods section, what was used as mock treatment. It is unclear, how much ethanol was applied to plants and whether pH was taken into account. For example, the pH of 1 mM SA should be about 3 - this is rather acidic and thus it is unclear, if the observed stomatal closure was due to SA or due to acid treatment.

We have clarified the methods (Lines 570-575). Regarding the pH of the treatments, we measured the pH of treatment and mock solutions for SA, JA, and ABA. Our mock solutions had low pH, e.g. SA mock pH 3.7 was similar to SA solution pH 3.5. SAs effect on stomatal aperture is well characterised in the literature and our control (and JA solution) treatments had more open stomata despite acidic pH.

Figure 5D, E. It is difficult to conclude whether ABA-treatment increased growth under

mite-infestation, because areas for control-treated plants are not shown. Please include control plant areas.

Thank you for your comment. We have added the control area and plant image to make it easier to perceive the ABA effect in Fig. 5DE.

Figure 7A, E. Would be good to show also true stomatal density values per unit leaf area, at least as a supplemental figure. At present, it seems something has gone rather wrong with these numbers, as numbers that give log10 values of 10-60 are extremely large.

We appreciate the referee's suggestion regarding the representation of the data. The plot has been updated to display the number of stomata per mm^2 and removed the log scale. Due to the possibility of a mistake in making the original panel, we also double-checked the numbers, which are high compared to the literature, but not unreasonable. Stomatal development is plastic and the higher SDs are likely due to specific plant growth conditions.

Table S4. Figure panel letters in the table do not match the figures. For example, there are only A, B C panels in Figure 1, but according to Table S4, there should also be D-G. Please correct.

We want to express our appreciation for your detailed review. Table S5 has been revised and updated based on your feedback.

General comments:
1) Overall comment on indicating statistical significance in figures - I think there is no reason to show both the p-value and asterisks. The p-value alone is sufficient and more informative than the asterisks, so I suggest to show only p-values. Further, if there is a specific p-value and not a comparison with a significance threshold, such as 0.0021 in Fig 1C, I suggest to state $p = 0.0021$, not $p < 0.0021$.

We appreciate your expertise and attention to detail in reviewing the statistics. Representation of significance has been modified. Asterisks have been removed and your note on 'P =' vs 'P <' has also been also corrected.

2) I also suggest to consider using Tukey post hoc test after two-way ANOVA instead of the Student-Newman-Keuls test, as the latter is more prone to type I errors and is usually not recommended when more than three groups' means are compared. The Methods section even says that Tukey test has been used, but this does not match the legends.

Thank you there was a mistake when writing the figure legends. The Tukey test was used, as stated previously in the methods and we have corrected the legends and checked to ensure that all statistical tests are correctly reported.

3) I suggest adding sample sizes to each figure legend.

As per your suggestions, the figure legends have been carefully reviewed and modified accordingly.

4) I suggest to revise the tables with respect to the precision and significant digits of the presented numbers - for example, in Table S2, if the mean is presented with one digit after decimal point, presenting the measures of errors with three does not make sense. It is also questionable, whether 4 significant digits are justified for the precision of stomatal aperture measurements presented in Table S1.

Your input has been incorporated into the tables to refine the work.

5) Although several legends say that single factors' (e.g. infestation time and mite treatment; or mite treatment and nucleus type) significance is indicated by R and C, these are not shown in the figures, only interaction effects are shown. Showing only significant interaction is fine, but the legends are confusing, if presented this way.

We have now included all significant factors in each figure. We apologise for this oversight.

6) Plant infestation: it is not clear, which experiments used whole plant and which used detached leaves, would be good to include this in figure legends.

Materials and methods have been revised and the infestation methodology has been added to every figure accordingly.

Reviewer #2 (Comments for the Author):

In the Ms, authors explored Arabidopsis response to mite T. urticae infestation and established the role of ABA in stomatal closure to effectively hinders mite infestation. The study is intresting, moreover the role of ABA is already well established in stomata defence in water stress. This study provides an interesting relation between stomatal aperture and mite infestation.
Introduction is nicely written with the updated information and gap areas. Authors found that the accumulation of SA, JA, JA-Ile and ABA was increased after mite infestation, especially the ABA. Further, they found a correlation between ABA, stomatal closure and mite infestation by using numerous approaches including various ABA related mutants analysis, exogenous application of ABA etc.

It seems to be a complete study, moreover, it would be more interesting if authors may include some mechanistic details.

Thank you for your helpful feedback. We appreciate very much the reviewer´s positive comments and acknowledgement of the completeness of our study. We have added several mechanistic paragraphs to the manuscript on the reviewer's recommendation, as detailed below.

How mite infestation induces the ABA accumulation should be included. Besides, authors analysed the ROS, but usually ROS and calcium signalling are related to each other, what is the status of Calcium in cell may also be checked.

We thank the reviewer for this comment. We have added discussion text re-examining our previous transcriptomic analysis of mite infestation (Santamaria et al. 2021; J Integr Plant Biol 33, 312) and how ABA biosynthetic, catabolic, deconjugation and transport processes may establish and coordinate the ABA pattern (Lines 408-432). The strong modulation of these processes, along with their mutant phenotypes, support ABA's role in the defence mechanism and offers many opportunities for future investigation, particularly BGLU18's dual role in defence compound production and ABA accumulation.

Indeed, ROS and calcium signalling show interesting interactions under a variety of stresses including herbivory. We have added a text, discussing the potential interaction (line 442-445). Future work analysing Ca^{2+} levels would help to understand this

relationship and maybe to clarify stomata/ABA function. We feel that potential calcium/ROS/ABA synergistic crosstalk is complex, and as we show that ROS were correlated with mite feeding and anticorrelated with ABA, we feel this is beyond the scope of the current work.

Authors analysed the ABA biosynthesis and catabolism related mutants aba2-1 and cyp707a1cyp707a3, along with e 112458 ABA receptor sextuple mutant and ost1-3 SnRK2 kinase mutant. But, they have not studied any sensor elements. ABA usually functions downstream or slightly late responsive. It will be interesting if authors identify the sensor protein which first recognizes the mite infestation, and then induces the ABA biosynthesis and further downstream signalling.

We have added a section to the discussion (Lines 396-407) discussing potential signals, perception and signaling upstream of ABA accumulation. This includes discussion of mechanical damage which can cause ABA accumulation and Tet1 and Tet2 which are strong candidates as HAMPs and can induce ABA accumulation. However, there may be several further signals. Similarly, which PRRs detect mite herbivory upstream of ABA accumulation remains to be established. Identification and characterisation of these events is beyond the scope of this work.

The title also not seems to be appealing.

We have changed the title to '**Spider mite feeding induces an abscisic acid dependent stomatal defense.'**

08-Jan-2024

Dr. Alexander M Jones
Sainsbury Laboratory Cambridge University
Sainsbury Laboratory
Cambridge, Please select an option below CB2 1LR
United Kingdom

RE: Spider mite herbivory induces an abscisic acid-driven stomatal defense

Dear Dr. Alexander Jones:

We are pleased to accept your manuscript for publication in the Plant Physiology. This acceptance is contingent on revision based on the comments of one reviewer and myself. In particular, please consider the following minor points raised below by the referee

Please highlight all changes and include a detailed annotation to changes to the text, with line numbers, and noting your responses to the comments.

To submit your revised manuscript, click:

Link Not Available

If you cannot return the revised manuscript within 8 weeks of receipt, please let us know. Otherwise, we will assume that you have elected not to revise the manuscript and are withdrawing it.

Thank you for allowing us to review your work. We look forward to hearing from you soon.

Sincerely,

Board of Editors, Plant Physiology

-- Reviewer comments:
Reviewer #1 (Comments for the Author):

I thank the authors for taking on board all my comments in the revised version of the manuscript. I still have very few minor comments, specifically:

Line 242 - reference to figure S2A does not seem to be appropriate, should be 4A?

Line 244 - reference to figure S2B does not seem to be appropriate, should be 4C?

Line 253 - reference to figure S2C,D does not seem to be appropriate, should be 4B,D?

Line 264 - reference to figure S4B does not seem to be appropriate, should be S2B?

Figure S3A - SF-s seem to be missing from the figure

Overall, I think this is a really exciting paper and I enjoyed reading it very much. When I read the manuscript the first time in previous round of review, I was sceptical to start with, but the data presented in the manuscript convinced me that indeed, stomatal numbers and apertures are important during mite infestation. This opens up exciting new avenues of research and potentially, applications, and I am looking forward to seeing how this research will be taken further in follow-up studies.

Please find below our response to reviewers.

--- Reviewer comments:

Reviewer #1 (Comments for the Author):

I thank the authors for taking on board all my comments in the revised version of the manuscript. I still have very few minor comments, specifically:

Line 242 - reference to figure S2A does not seem to be appropriate, should be 4A?

Corrected

Line 244 - reference to figure S2B does not seem to be appropriate, should be 4C?

Corrected

Line 253 - reference to figure S2C,D does not seem to be appropriate, should be 4B,D?

Corrected

Line 264 - reference to figure S4B does not seem to be appropriate, should be S2B?

Corrected

Figure S3A - SF-s seem to be missing from the figure

Amended, as well as an identical error in S4, these were actually single factor ANOVAs, and so shouldn't have the SFs, we have corrected the legends to reflect this.

Overall, I think this is a really exciting paper and I enjoyed reading it very much. When I read the manuscript the first time in previous round of review, I was sceptical to start with, but the data presented in the manuscript convinced me that indeed, stomatal numbers and apertures are important during mite infestation. This opens up exciting new avenues of research and potentially, applications, and I am looking forward to seeing how this research will be taken further in follow-up studies.

Thank you so much for reviewing our paper. Your kind words on the work have been very encouraging and your diligent reviewing has been very useful to us, producing a much improved manuscript.

14-Mar-2024
Dr. Alexander M Jones
Sainsbury Laboratory Cambridge University
Sainsbury Laboratory
Cambridge, Please select an option below CB2 1LR
United Kingdom

MSID: PP2023-RA-01949DR1
MS TITLE: Spider mite herbivory induces an abscisic acid-driven stomatal defense

Dear Dr. Alexander Jones:

I am pleased to inform you that your revised manuscript "Spider mite herbivory induces an abscisic acid-driven stomatal defense" has been accepted for publication in Plant Physiology. Your manuscript will be checked for consistency with journal formatting and image standards by one of our Science Editors, who will contact you shortly. They may have a number of queries and suggestions for you. Please keep in mind that they work to increase the online visibility of your article and its attraction for readers from the widest of backgrounds. You will have the opportunity to review any revisions, and I hope you will work constructively with them to get the most out of your research. You will also have the opportunity to review all changes when you receive the author proofs for your article.

Once validated for publication, your article will be sent to a Science Editor for a final review of your manuscript and source files. After your final files are uploaded, your article will appear in the next available issue and the online Preview version of your article will be posted after your signed Copyright and Checklist forms and final source files are received.

Please be sure to include the following in your manuscript file:
Harvard style citations
List of all author contributions and funding information at the beginning of manuscript after the titles and author list.
List all supplemental materials associated with the article, with titles, at the end of the ms, before the references and acknowledgments section.
Complete Figure Legends for all figures appearing in the article listed before the Literature Cited.

Thank you and congratulations on your Plant Physiology paper!

Sincerely,
Board of Editors, Plant Physiology

=====
FIRST AUTHOR PROFILES
=====

We recognize the hard work that goes into being First Author of a Plant Physiology paper by publishing a "First Author Feature" for each First Author alongside your paper.

If the first author(s) would like to submit information for a feature on Facebook and Twitter, fill out the form by clicking on the link below:

Plant Physiology First Author Feature Form: https://forms.office.com/r/ZBCGZfhm8F
If you cannot access the form through the link above, please contact Rachel at rbelsky@aspb.org.

To upload an image of yourself to accompany your profile, please put your photo in the correct journal folder at the link below. You must label your image: Author First Name Last Name Article Title.

First Author Profile Image Dropbox: https://www.dropbox.com/scl/fo/xz029p7qjr2jml2rp2kmq/h?dl=0&rlkey=6fkxcaqmxx35ap152b2osy1fz

=====

IMPORTANT REMINDER: PEER REVIEW REPORTS
=====

If you opted to publish a peer review report along with your article during the original submission process, it will be prepared by the editorial staff and publicly posted with your manuscript, inside the zip file that contains any other supplemental material. As a reminder, the peer review report is a public record of all comments from editors and reviewers, as well as your prior responses, as you received them in the decision letters for each draft of your manuscript. If you agreed to publish this report and have changed your mind, or are not sure if you selected this option, please contact the editorial office as soon as possible before signing the license agreement from our publisher.

-- Reviewer comments:
